# Enhanced S-Cone Syndrome: Spectrum of Clinical, Imaging, Electrophysiologic, and Genetic Findings in a Retrospective Case Series of 56 Patients

**DOI:** 10.1016/j.oret.2020.07.008

**Published:** 2021-02

**Authors:** Emanuel R. de Carvalho, Anthony G. Robson, Gavin Arno, Camiel J.F. Boon, Andrew A. Webster, Michel Michaelides

**Affiliations:** 1 Moorfields Eye Hospital, London, United Kingdom; 2 Department of Ophthalmology, Amsterdam University Medical Centers, Amsterdam, The Netherlands; 3 UCL Institute of Ophthalmology, University College London, London, United Kingdom; 4 Department of Ophthalmology, Leiden University Medical Center, Leiden, The Netherlands

**Keywords:** Electroretinography, Enhanced S-cone syndrome, Molecular genetics, *NR2E3*, BCVA, best-corrected visual acuity, CME, cystoid macular edema, DA, dark-adapted, ESCS, enhanced S-cone syndrome, FAF, fundus autofluorescence, ISCEV, International Society for Clinical Electrophysiology of Vision, LA, light-adapted, logMAR, logarithm of the minimum angle of resolution, PERG, pattern electroretinography, RPE, retinal pigment epithelium, SD, standard deviation, SEM, standard error of the mean

## Abstract

**Purpose:**

To describe the detailed phenotype, long-term clinical course, clinical variability, and genotype of patients with enhanced S-cone syndrome (ESCS).

**Design:**

Retrospective case series.

**Participants:**

Fifty-six patients with ESCS.

**Methods:**

Clinical history, examination, imaging, and electrophysiologic findings of 56 patients (age range, 1–75 years) diagnosed with ESCS were reviewed. Diagnosis was established by molecular confirmation of disease-causing variants in the *NR2E3* gene (n = 38) or by diagnostic full-field electroretinography findings (n = 18).

**Main Outcome Measures:**

Age at onset of visual symptoms, best-corrected visual acuity (BCVA), quantitative age-related electrophysiologic decline, and imaging findings.

**Results:**

Mean age at onset of visual symptoms was 4.0 years, and median age at presentation was 20.5 years, with mean follow-up interval being 6.1 years. Six patients were assessed once. Disease-causing variants in *NR2E3* were identified in 38 patients. Mean BCVA of the better-seeing eye was 0.32 logarithm of the minimum angle of resolution (logMAR) at baseline and 0.39 logMAR at follow-up. In most eyes (76% [76/100]), BCVA remained stable, with a mean BCVA change of 0.07 logMAR during follow-up. Nyctalopia was the most common initial symptom, reported in 92.9% of patients (52/56). Clinical findings were highly variable and included foveomacular schisis (41.1% [26/56]), yellow-white dots (57.1% [32/56]), nummular pigmentation (85.7% [48/56]), torpedo-like lesions (10.7% [6/56]), and circumferential subretinal fibrosis (7.1% [4/56]). Macular and peripheral patterns of autofluorescence were classified as (1) minimal change, (2) hypoautofluorescent (mild diffuse, moderate speckled, moderate diffuse, or advanced), or (3) hyperautofluorescent flecks. One patient showed undetectable electroretinography findings; quantification of main electroretinography components in all other patients revealed amplitude and peak time variability but with pathognomonic electroretinography features. The main electroretinography components showed evidence of age-related worsening over 6.7 decades, at a rate indistinguishable from that seen in unaffected control participants. Eighteen sequence variants in *NR2E3* were identified, including 4 novel missense changes.

**Conclusions:**

Enhanced S-cone syndrome has a highly variable phenotype with relative clinical and imaging stability over time. Most electroretinography findings have pathognomonic features, but quantitative assessment reveals variability and a normal mean rate of age-related decline, consistent with largely nonprogressive peripheral retinal dysfunction.

Enhanced S-cone syndrome (ESCS; Online Mendelian Inheritance in Man identifier, 268100) is an autosomal-recessive retinal dystrophy caused by disease-causing variants in nuclear receptor subfamily 2, group E, member 3 (*NR2E3*), a member of the nuclear hormone receptor superfamily of ligand-modulated transcription factors, also known as photoreceptor-specific nuclear receptor (Online Mendelian Inheritance in Man identifier, 604485).^1^, ^2^, ^3^, ^4^ Goldmann-Favre syndrome has also been shown to be caused by biallelic variants in *NR2E3*, rendering distinction between the 2 entities redundant.^5^, ^6^, ^7^, ^8^, ^9^ Similarly, recessive variants in *NR2E3* have been described in cases of clumped pigmentary retinal degeneration.^6^ A single missense *NR2E3* variant (p.G56R) has also been linked to autosomal-dominant retinitis pigmentosa (Online Mendelian Inheritance in Man identifier, 611131).^10^^,^^11^

*NR2E3* was identified first by its homology to *NR2E1*, which acts on cell-fate determination in *Drosophila* and encodes an orphan receptor of the steroid and thyroid hormone receptor superfamily of ligand-activated transcription factors.^12^^,^^13^ In the eye, *NR2E3* regulates the fate of retinal progenitor cells during retinogenesis.^14^, ^15^, ^16^, ^17^ The different cell subtypes in the vertebrate retina derive from a common population of multipotent progenitors.^18^^,^^19^ Cone primordial cells arise earlier than rod cells.^16^^,^^20^ Cellular interactions between cones dictate the ensuing spatial rearrangement, opsin expression, and ratio of photoreceptor subtypes in the mature retina. Disease-causing variants in *NR2E3*, expressed in late retinal progenitors and differentiating photoreceptors in the outer nuclear layer of the retina, disrupt the determination of photoreceptor cell fate, affecting the normal ratio and topographic distribution of the different photoreceptor subtypes in the mature retina.^21^^,^^22^ S-cones are expressed earlier than M-cone (medium-wavelength) and L-cone (long-wavelength) photoreceptors and are therefore regarded as the default primordial cone cells.^22^ As a result, in the absence of *NR2E3*, rods develop into nonfunctional hybrid photoreceptors and L-cone and M-cone expression is suppressed with a concomitant overexpression of ancestral S-cones.^15^^,^^16^^,^^20^^,^^22^, ^23^, ^24^, ^25^

The unique photoreceptor arrangement in patients harboring *NR2E3* variants is responsible for the increased sensitivity to blue light^26^ and often reflected by pathognomonic full-field electroretinography responses. The dark-adapted (DA) rod-specific dim flash (DA 0.01) electroretinography features are typically undetectable, although detectable responses have been reported in mild ESCS, which has been suggested to stem from functional dimerization of *NR2E3* mediated by ligand-binding domain variants.^27^, ^28^, ^29^ Responses in the retina are dominated by short-wavelength–sensitive mechanisms, leading to a similar simplified and severely delayed waveform under DA and light-adapted (LA) conditions, with a severely abnormal LA 30-Hz flicker electroretinography response.^21^^,^^30^, ^31^, ^32^, ^33^ Short-wavelength–specific stimulation may elicit a high-amplitude response when compared with those of normal participants, consistent with the increased number of S-cone photoreceptors.^23^

Previously reported symptoms of patients with ESCS include nyctalopia, variable visual acuity loss, and constricted field of vision.^33^, ^34^, ^35^ The clinical signs encompass a combination of yellow-white dots, nummular pigmentation at the level of the retinal pigment epithelium (RPE), especially along the temporal vascular arcades, and variable degrees of foveomacular schisis.^31^^,^^33^^,^^35^, ^36^, ^37^, ^38^, ^39^, ^40^ Other signs include torpedo-like retinal lesions, cystoid macular edema, and circumferential subretinal fibrosis, the latter thought to occur secondary to choroidal neovascularization.^41^^,^^42^ Although clinical and electroretinographic characteristics are well recognized, published analyses are often qualitative, and data relating to the natural history of the disorder are lacking. The purpose of the present study was to review clinical and electrophysiologic data of a large cohort of patients diagnosed with ESCS retrospectively to define better variability of the phenotype, long-term visual outcomes, severity and stability of retinal dysfunction, and the nature of *NR2E3* disease-causing variants.

## Methods

A cohort of 56 patients with a clinical diagnosis of ESCS were ascertained at Moorfields Eye Hospital (n = 45) and the Expertise Center for Hereditary Retinal Diseases of Amsterdam University Medical Centers/Leiden University Medical Center (n = 11), with a mean follow-up of 6.1 years (range, 0–34 years). All patients were diagnosed first between 1984 and 2018, with the latest examination performed in 2019. A baseline electroretinography examination was performed in 31 patients and repeated in 3 patients. The cohort included 3 cases of pseudodominance with consanguineous parents, and 5 sibships (4 sibling pairs, 1 sibling pair with an affected parent, and 2 pairs of 1 affected parent and 1 affected child). Molecular confirmation of the diagnosis was established in 43 patients, and 24 of these underwent baseline electroretinography. The diagnosis was established on pathognomonic electroretinography responses and phenotypical retinal changes in the remaining 13 patients. The protocol of the study adhered to the tenets of the Declaration of Helsinki and was approved by the ethics committee of all involved institutions, namely Moorfields Eye Hospital, University Medical Centre Amsterdam and Leiden University Medical Centre. Due to the retrospective nature of the study, informed consent was not required. Thirteen cases were described previously but without detailed electroretinography quantification and longitudinal data.^33^^,^^43^^,^^44^

### Clinical Assessment

Fifty-six patients were ascertained. Six patients were assessed on a single occasion and all others on at least 2 occasions. In the latter group, the initial and last visits were considered as baseline and follow-up examinations, respectively. Follow-up time was determined by the interval between age at baseline and age at the latest follow-up examination.

Color contrast sensitivity was assessed in 12 patients along tritan, protan, and deutan axes using the ChromaTest, involving the use of colored optotypes presented on a randomized luminance noise background.^45^^,^^46^ In all patients, a medical history was obtained and a comprehensive ophthalmologic examination was performed that included best-corrected Snellen visual acuity converted to equivalent logarithm of minimal angle of resolution (logMAR) for the purpose of data analysis.^47^ Retinal fundus photographs were obtained by conventional 35° fundus color photography (Topcon Europe Medical BV, Capelle aan den IJssel, The Netherlands) or wide-field confocal scanning laser imaging (Optos PLC, Dunfermline, United Kingdom). Spectral-domain OCT (Heidelberg Engineering, Heidelberg, Germany) macular scans were performed in all patients.

The patterns of macular and peripheral fundus autofluorescence (FAF) were assessed in 49 pairs of eyes. Macular FAF images were obtained using a confocal scanning laser ophthalmoscope with blue-light excitatory beam (Spectralis; Heidelberg Engineering). When available, peripheral FAF images were analyzed with wide-field Optos imaging with green-light excitatory beam. Specific macular and peripheral FAF patterns were classified as: (1) no change; (2) hypoautofluorescence—minimal change pattern, mild diffuse, moderate speckled, moderate diffuse (mid-peripheral half-ring or ring ≤5000 μm in widest diameter), moderate diffuse (more than 5000 μm hypoautofluorescence), or nummular (patchy); and (3) hyperautofluorescent flecks.

### Electrophysiologic Assessment

A total of 32 patients underwent electrophysiologic assessment at Moorfields Eye Hospital (age range, 6–73 years at the time of testing). The electrophysiologic assessment included full-field electroretinography and pattern electroretinography (PERG), incorporating the minimum standards of the International Society for Clinical Electrophysiology of Vision (ISCEV)^48^^,^^49^ and recorded using gold foil corneal electrodes. Additionally, 28 patients underwent short-wavelength flash electroretinography (S-cone electroretinography),^50^ obtained using a blue stimulus (445 nm, 80 cd/m^2^; stimulus duration, 5 ms) on a constant orange background (620 nm, 560 cd/m^2^).^33^ S-cone electroretinography peak times were measured to the second or single positive peak; amplitudes were measured from baseline to the single or second positive peak^50^ or, if an early negative trough was present, as a trough-to-peak amplitude to better characterize the magnitude of responses.

The patient data were compared with the control (normative) electrophysiologic data obtained from 160 healthy participants (age range, 10–79 years), which included validated recordings for DA rod-specific dim flash (DA 0.01; n = 117), DA 10.0 (n = 141), LA 3.0 30-Hz (n = 131), and the LA 3.0 (single flash cone) electroretinography (n = 109). The amplitude and peak time ratios between the LA 3.0 electroretinography a-wave and LA 30-Hzelectroretinography were calculated for each patient, and these and other main electroretinography components were compared with age and the control data.

A total of 3 patients seen at the Amsterdam University Medical Centers underwent baseline electrophysiologic assessment. Electrophysiologic data concerning these patients were excluded from analysis, given that flash electroretinography examinations were performed according to older or abbreviated protocols using silver thread electrodes, precluding comprehensive electroretinography phenotyping and direct comparison with ISCEV-standard recordings.

### Genetic Screening

Patients were screened for disease-causing variants by direct sequencing of all 8 exons and intron–exon boundaries of *NR2E3*. Subsequently, available relatives also underwent sequencing. Genomic DNA was isolated from peripheral blood lymphocytes using a kit (Gentra Puregene blood extraction kit; Qiagen, Hilden, Germany). DNA was amplified using specifically designed primers by polymerase chain reaction, and the polymerase chain reaction fragments were sequenced using standard protocols (details are available from the authors on request). The likely pathogenicity of novel missense variants was assessed using the predictive algorithms of InterVar (according to the guidelines by the American College of Medical Genetics and Genomics and the Association for Molecular Pathology for interpretation of causality of sequence variants; http://wintervar.wglab.org),^51^ the Protein Variation Effect Analyzer (http://provean.jcvi.org/index.php),^52^, ^53^, ^54^ and PolyPhen-2 (http://genetics.bwh.harvard.edu/pph2).^55^ Information regarding the domain structure of *NR2E3* was retrieved using UniProtKB-Q9Y5X4 (NR2E3_HUMAN). To predict the consequences of the ligand-binding domain missense mutations in the 3-dimensional space, we analyzed the crystal structure of apo *NR2E3* ligand-binding domain with pdb code 4LOG, retrieved from the SWISS-MODEL server.^56^, ^57^, ^58^, ^59^

### Statistical Analysis

The mean, standard error of mean, median, standard deviation, and range were used as appropriate. Best-corrected visual acuities (BCVAs) were ascertained and converted to logarithm of the minimum angle of resolution (logMAR) scale for statistical analysis.^47^ Mean BCVA change over follow-up was calculated per each eye, right and left, using the related samples Wilcoxon signed-rank test with *P* < 0.05 deemed clinically significant. Variability between BCVA in the right and left eye recordings at baseline and follow-up was assessed using the paired samples Wilcoxon signed-rank test, with *P* < 0.05 deemed clinically significant. Age was correlated with BCVA at baseline and follow-up applying a Spearman correlation model with *P* < 0.05 deemed clinically significant. The relationship between visual acuity and electrophysiologic responses was assessed by multiple linear regression analysis, and *P* < 0.05 was considered statistically significant. Pearson correlation coefficients were calculated to compare the PERG P50 measure of macular function with central visual acuity and age. Photopic electroretinography a-wave-to-30-Hz flicker ratios were compared with the unpaired Mann–Whitney *t* test. Statistical analyses were performed using IBM SPSS software version 25.0 (IBM Corp, Armonk, NY) and GraphPad Prism software version 8 (GraphPad Software, San Diego, CA).

## Results

### Clinical Findings

Fifty-six patients, including 33 female patients (59%) and 23 male patients (41%), were included. Thirty patients were of European origin (54%), 20 patients were of Middle Eastern origin (36%), 4 patients were of South Asian origin (7%), and 2 patients were of Black origin (3%). Nyctalopia was reported as the first symptom in 52 patients (93%), with or without reduced central visual acuity. The remaining 4 patients described reduced central acuity without nyctalopia as the initial symptom. At presentation, a manifest squint was observed in 9 patients and nystagmus was recorded in 3 patients. Refractive assessment was conducted in 21 patients, hyperopia in 12 patients (57.1%), myopia in 6 patients (28.5%), and plano in 3 patients (14.2%). Color contrast sensitivity was tested in 9 patients; 6 patients showed relative sparing of the tritan axis and moderately elevated protan and deutan thresholds, 1 patient showed nonspecific dyschromatopsia, and 2 patients showed normal results.

The median age at onset of visual symptoms was 4.0 years (range, 0–27 years). The median ages at presentation to the eye clinic (baseline) and follow-up were 20.5 years (range, 1–75 years) and 33.0 years (range, 2–81 years), respectively. The mean follow-up interval was 6.1 years (range, 0–34 years). Six patients were assessed once. Twenty-eight patients (50%) sought treatment before 21 years of age. Thirteen patients sought treatment after 50 years of age (23%).

**Figure 1:**
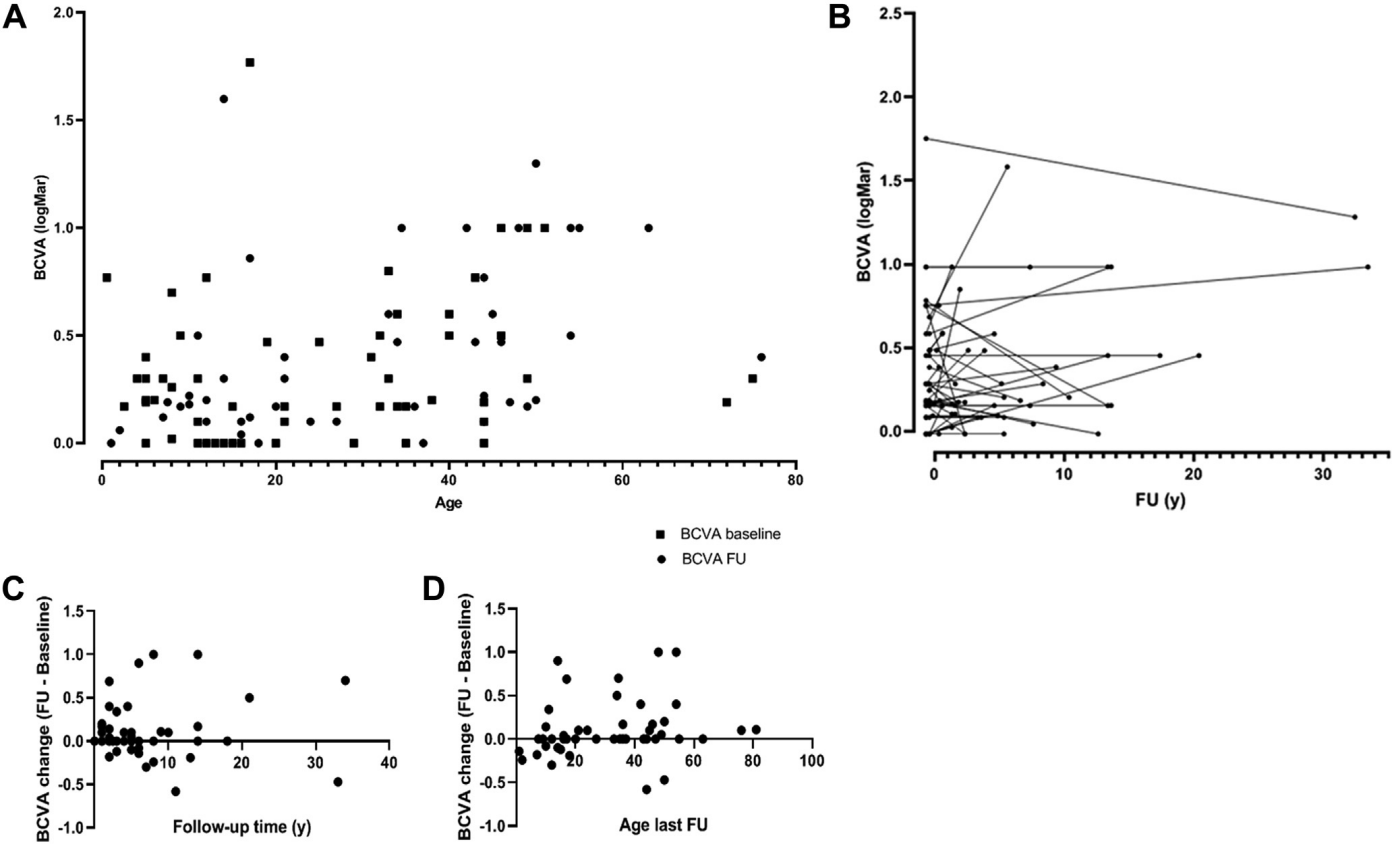
**A**, Plot showing best-corrected visual acuity (BCVA; logarithm of the minimum angle of resolution [logMAR]) of the right eye at baseline and last follow-up visit against the patient’s age. **B**, Plot showing BCVA (logMAR) in the right eye as a function of period of follow-up time per individual patient. **C**, **D**, Plots showing BCVA change in the right eye (BCVA_FU_ – BCVA_baseline_) as function of (**C**) follow-up (FU) time (y = years) and (**D**) age at baseline.

The mean BCVAs of the better-seeing eye at baseline and follow-up were 0.32 logMAR (standard error of the mean [SEM], 0.045 logMAR; range, 0.0–1.77 logMAR) and 0.39 logMAR (SEM, 0.054 logMAR; range, 0.0–1.60 logMAR), respectively (Table 1). No clinically significant difference was found between BCVA in the right and left eyes (*P* = 0.14 for baseline BCVA, *P* = 0.22 for follow-up BCVA). Overall, mean BCVA change was 0.01 logMAR (SEM, 0.05 logMAR), although this included patients with short follow-up time (<6 years). In the group with extended follow-up time (≥6 years; n = 21) mean BCVA change was 0.12 logMAR (SEM, 0.09 logMAR; Fig 1). A score of BCVA severity was attributed for each eye, and progression over time was assessed for each eye separately. Severity was graded as very mild (BCVA, ≤0.1 logMAR; n = 7 [12.5%]), mild (≤0.3 logMAR; n = 21 [37.5%]), moderate (≤0.6 logMAR; n = 12 [21.4%]), severe (≤1 logMAR; n = 11 [19.6%]), and very severe (>1.0 logMAR; n = 5 [9%]). No progression was observed in 79 of 100 eyes. Ten eyes progressed from very mild and mild severity scores to moderate severity (10%). Five eyes progressed from mild and moderate severity scores to severe scores (5%), and 6 eyes progressed from severe to very severe scores (6%). Poor visual acuity (BCVA, >0.6 logMAR) was observed in 16 patients at baseline (28.5%; median age, 29.0 years; range, 0.5–51 years) and in 18 patients at last follow-up (36%). In 2 patients, BCVA loss in 1 eye could be attributed directly to other significant ophthalmic events, namely retinal detachment and dense amblyopia. In 5 other patients, BCVA loss could be ascribed partly to concurrent ophthalmic pathologic features. In 1 patient with undetectable cone and rod electroretinography responses, optic disc pallor was observed at initial presentation. The patient was assessed by neuro-ophthalmology and no underlying neuro-ophthalmic cause was found for the optic neuropathy. In 6 patients with documented BCVA worsening over time, no other unrelated significant ophthalmic events were reported. Most patients (13/18) with severe visual outcomes showed moderate to advanced foveomacular schisis, with accompanying or ensuing macular atrophy in 8 patients. Three patients demonstrated advanced macular atrophy at the initial visit. Cystoid macular edema was confirmed on fluorescein angiography in 1 patient and diagnosed based on structural OCT appearance in 3 other patients with severe visual outcomes. Treatment with oral carbonic anhydrase inhibitors was attempted in 4 patients, with positive anatomic responses attained in 2 patients, albeit with no visual improvement noted after treatment. One patient was diagnosed with congenital nystagmus at birth, with delayed visual development that might have contributed to poor visual outcome. No significant correlation was found between PERG responses and clinical severity. Clinical findings are summarized in Tables 1, 2, and 3.

**Table 1:** Clinical Data and Molecular Genetic Status of 56 Patients with Enhanced S-Cone Syndrome

Patient No.	Family Identification	Age (yrs)			Length of Follow-up (yrs)	Visual Acuity (Logarithm of the Minimum Angle of Resolution)		Variants Identified
		Onset	Baseline	Follow-up		Baselin	Follow-up	
1	NA	0	49	63	14	1/1	1/1	c.119-2A→C (Hom)
2	19824	4	11	16	5	0.1/0.6	0.1/0.6	c.119-2A→C/c.1194delT; p.P399Qfs∗3
3	19824	0	9	14	5	0.5/0.5	0.4/0.3	c.119-2A→C/c.1194delT; p.P399Qfs∗3
4	19940	3	15	17	2	0.17/0.6	0.86/1.2	c.305C→A; p.A102D/c.767C→A; p.A256E
5	20195	3	8	14	6	0.7/0.7	1.6/1.6	c.119-2A→C/c.1194delT; p.P399Qfs∗3
6	20766	4	44	49	5	0.12/0	0.17/0.17	**c.211T**→**C; p.F71L**/c.932G→A; p.R311Q
7	22497	5	8	11	3	0.26/0.36	0.6/0.5	NA
8	16200	6	34	48	14	0.77/0.6	1.77/1	c.119-2A→C (Hom)
9	23115	2	7	8	2	0.3/0.5	0.3/0.17	c.119-2A→C/c.1025T→C; p.V342A
10	27357	6	2.5	20	14	0.17/0.17	0.17/0.18	c.311G→A; p.R104Q (Hom)
11	15494	10	17	50	33	1.77/1.77	1.3/1.3	c.311G→A; p.R104Q (Hom)
12	27357	5	5	18	13	0.19/0.19	0/0	c.311G→A; p.R104Q (Hom)
13	3006	0	32	46	14	0.3/0.17	0.47/0.47	c.119-2A→C/c.767C→A; p.A256E
14	4644	1	13	34	21	0/0.47	0.5/0.47	c.119-2A→C (Hom)
15	16337	20	25	43	18	0.47/0.77	0.47/0.77	c.119-2A→C/c.932G→A; p.R311Q
16	15128	0	0.5	34.5	34	0.77/0.77	1.47/1	c.119-3C→G (Hom)
17	NA	6	21	35	14	0.17/2	0.17/2	NA
18	18491	20	27	35	8	0.17/0.17	0.17/0.18	c.932G→A; p.R311Q/c.1112T→G; p.L371W
19	NA	12	34	37	3	0.3/0.17	0.3/0	NA
20	20091	0	40	42	2	0.6/0.6	1/1	NA
21	18411	27	43	44	1	1/0.77	1/0.7	NA
22	NA	5	12	No follow-up	No follow-up	0.9/0.77	No follow-up	NA
23	20907	0	5	7.5	2.5	0.3/0.3	0.3/0.19	NA
24	19784	0	5	1	6	0.14/0	0/0	NA
25	18115	4	44	47	3	0.19/0.19	0.19/0.19	c.646G→A; p.G216S (Hom)
26	18758	4	72	81	9	0.19/0.2	0.3/0.3	c.305C→A; p.A102D (Hom)
27	NA	4	12	15	3	0.12/0	0/0	NA
28	22924	4	11	12	1	0/0	0/0	c.932G→A; p.R311Q/c.747+1G→C
29	27135	5	35	36	1	0/0.17	0.17/0.3	NA
30	22633	4	15	17	2	0.12/0	0.12/0.22	c.932G→A; p.R311Q (Hom)
31	19530	4	14	16	2	0/0	0.04/0.06	NA
32	23064	5	5	7	2	0.3/0.2	0.12/0.12	c.310C→T; p.R104W (Hom)
33	19530	4	8	10	2	0.04/0.02	0.18/0.2	NA
34	24703	12	19	21	2	0.47/1.17	0.30/0.80	c.310C→T; p.R104W (Hom)
35	19668	5	5	12	7	0.6/0.4	0.3/0.2	c.311G→A; p.R104Q/c.767C→A; p.A256E
36	18880	0	46	54	8	1/1	1/1	c.119-2A→C/**c.908T**→**C; p.L303P**
37	NA	20	40	45	5	0.5/0.8	0.6/1	NA
38	17494	4	20	24	4	0/0	0.1/0.2	NA
39	NA	3	11	21	10	0.3/0.3	0.4/0.4	c.119-2A→C (Hom)
40	NA	11	44	No follow-up	No follow-up	0.3/0.1	No follow-up	c.932G→A; p.R311Q (Hom)
41	NA	4	33	44	11	0.8/1.1	0.22/1.7	c.119-2A→C (Hom)
42	NA	5	21	27	6	0.1/0.22	0.1/0.5	c.119-2A→C/c.932G→A; p.R311Q
43	NA	4	4	10	6	0.3/0.3	0.22/0.3	c.200_208del9del; p.C67_G69del (Hom)
44	NA	3	31	No follow-up	No follow-up	0.4/0.5	No follow-up	c.932G→A; p.R311Q/**c.739C**→**T; p.R247W**
45	NA	3	11	12	4	0.22/0.1	0.22/0.1	c.932G→A; p.R311Q (Hom)
46	NA	5	46	No follow-up	No follow-up	1/0.5	No follow-up	c.932G→A; p.R311Q (Hom)
47	NA	5	75	76	1	0.3/2	0.4/2	c.119-2A→C/c.227G→A; p.R76Q
48	25595	12	35	36	1	0.47/0.17	0.47/0.17	c.932G→A; p.R311Q (Hom)
49	25690	3	33	No follow-up	No follow-up	0.3/0.8	No follow-up	c.248G→A; p.C83Y (Hom)
50	23064	8	6	2	8	0.3/0.2	0.06/0.06	c.310C→T; p.R104W (Hom)
51	25574	10	49	50	1	0.3/0.3	0.5/0.2	c.119-2A→C (Hom)
52	247	10	38	54	4.5	0.6/0.2	1/0.5	c.119-2A→C/c.932G→A; p.R311Q
53	NA	16	16	No follow-up	No follow-up	0/0	No follow-up	**c.926G**→**A; p.R309Q** (Hom)
54	NA	25	29	No follow-up	No follow-up	0/0	No follow-up	**c.926G**→**A; p.R309Q** (Hom)
55	26532	0	32	33	1	0.6/0.5	0.6/0.6	c.119-2A→C (Hom)
56	22052	0	51	55	4	1/1	1/1	c.119-2A→C (Hom)

Hom = homozygous variant; NA = not available.

Putative novel changes appear in boldface.

**Table 2:** Clinical Characteristics of Patients with Severe Visual Impairment (Best-Corrected Visual Acuity, >0.6 Logarithm of the Minimum Angle of Resolution)

Patient No.	Age at Onset (yrs)	Length of Follow-up (yrs)	Visual Acuity (Logarithm of the Minimum Angle of Resolution)		Progression	Macular cHanges				Other Ophthalmic Pathologic Features
			Baseline	Follow-up		Foveomacular Schisis	Macular Edema	Carbonic Anydrase Inhibitor Treatment	Macular Atrophy	
1	0	14	1/1	1/1	N	N	N	N	Y	Senile cataracts
4	3	2	0.17/0.6	0.86/1.2	Y	Y	N	N	N	**Squint, amblyopia**
5	3	6	0.7/0.7	1.6/1.6	Y	N	Y	Y (no response)	N	
8	6	14	0.77/0.6	1.77/1	Y	Y	Y	Y (response)	Y	
11	10	33	1.77/1.77	1.3/1.3	N	Y	Y	Y (no response)	Y	
15	20	18	0.47/0.77	0.47/0.77	N	Y	Y	Y (response)	N	
16	0	34	0.77/0.77	1.47/1	Y	Y	N	N	Y (in one eye)	Congenital nystagmus
17	6	14	0.17/2	0.17/2	N	N	N	N	Y (in one eye)	**Amblyopia, retinal detachment (6 yrs of age)**
20	0	2	0.6/0.6	1/1	Y	N	N	N	N	Senile cataracts
21	27	1	1/0.77	1/0.7	N	Y	N	N	Y	
22	5	No follow-up	0.9/0.77	No follow-up	NA	Y	N	N	N	TED
34	12	2	0.47/1.17	0.30/0.80	N	Y	N	N	Y	
36	0	8	1/1	1/1	N	Y	N	N	Y	ERM, optic nerve pallor
37	20	5	0.5/0.8	0.6/1	Y	Y	N	N	Y	
41	4	11	0.8/1.1	0.22/1.7	N	Y	Y	N	N	
46	5	No follow-up	1/0.5	No follow-up	NA	Y	N	N	N	
49	3	No follow-up	0.3/0.8	No follow-up	NA	N	N	N	Y	
56	0	4	1/1	1/1	N	Y	N	N	Y	

ERM = epiretinal membrane; N = no; NA = not applicable; TED = thyroid eye disease; Y = yes.

Significant ophthalmic events that have contributed to poor visual acuity in 1 eye appear in boldface.

**Table 3:** Clinical Characteristics of the Enhanced S-Cone Syndrome Cohort

Characteristic	Data
No. of patients	56
Age at presentation (yrs)	
Median	4
Range	0–27
Age at first visit (yrs)	
Median	20.5
Range	1–75
Age at last follow-up (yrs)	
Median	33
Range	2–81
Length of follow-up (yrs)	
Mean	6.1
Range	0–34
BCVA (better-seeing eye) at presentation (logMAR)	
Mean	0.32
SEM	0.0–1.77
BCVA (better-seeing eye) at last visit (logMAR)	
Mean	0.39
SEM	0.0–1.6
BCVA reduction (logMAR)	
Mean	0.07
SEM	0.04
Gender, no. (%)	
Female	33 (58.9)
Male	23 (41.1)
Ethnicity, no. (%)	
White	30 (53.6)
Non-White	26 (46.4)
Refraction, no. (%)	
Plano	4 (7.1)
Myopia	5 (8.9)
Hyperopia	12 (21.4)
NA	35 (62.5)
First symptom/sign, no. (%)	
Nyctalopia	52 (92.9)
Squint	9 (16.1)
Nystagmus	3 (5.4)
Clinical signs, no. (%)	
Optic nerve pallor	3 (5.4)
Macular edema (based on structural OCT appearance)	16 (28.5)
Foveomacular schisis	23 (41.1)
Nummular pigmentation	48 (85.7)
Yellow dots	32 (57.1)
Circumferential subretinal fibrosis	4 (7.1)
Torpedo-like lesions	6 (10.7)
Vitreous opacities	12 (21.4)
Preretinal neovascularization	1 (1.8)

BCVA = best-corrected visual acuity; logMAR = logarithm of the minimum angle of resolution; NA = not specified; SEM = standard error of the mean.

### Clinical and Fundus Autofluorescence Features

**Figure 2:**
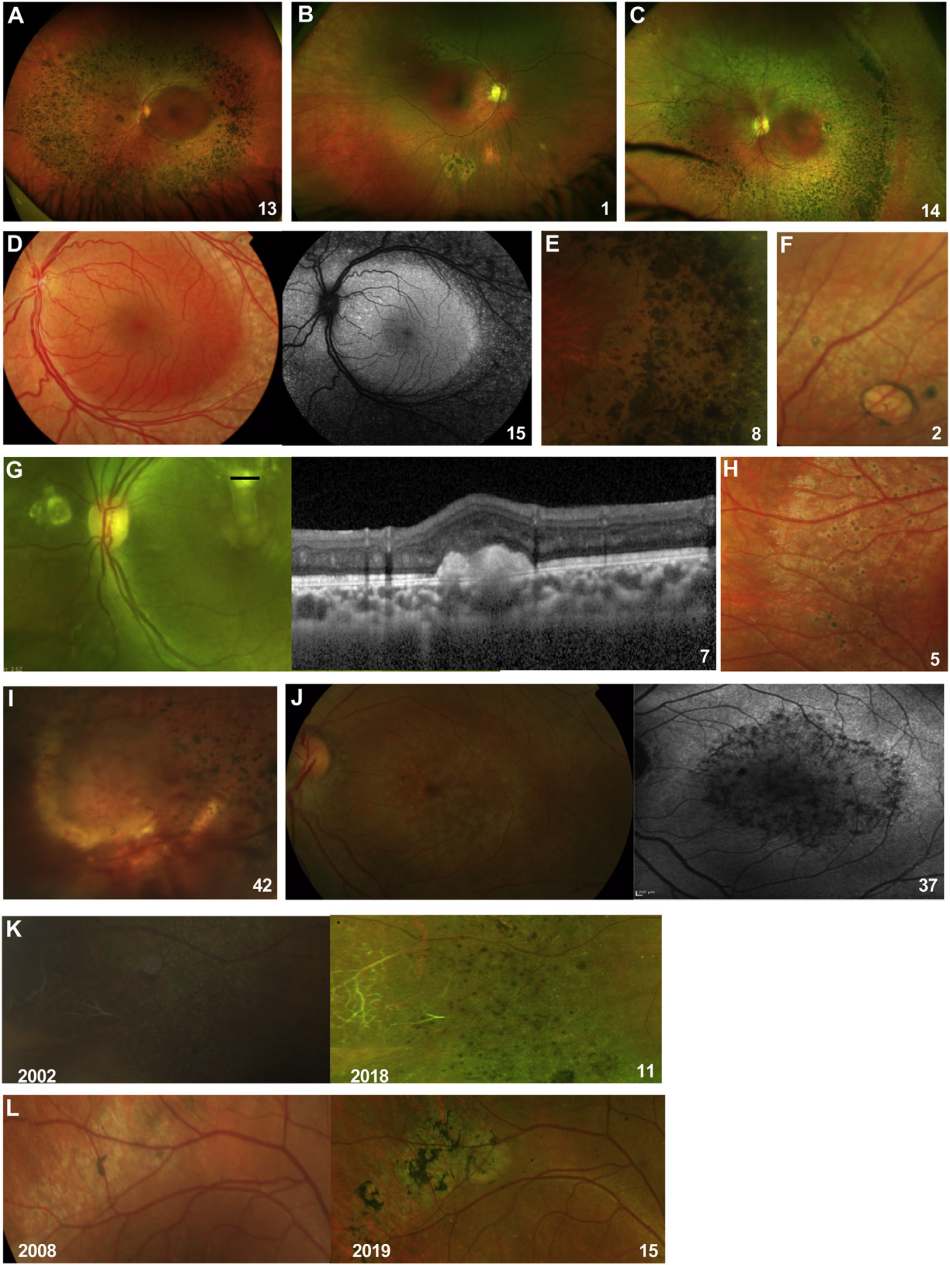
Images showing phenotypical variation of enhanced S-cone syndrome in individual patients (numbered). **A**, Nummular pigmentary deposition in the mid-peripheral retina. **B**, Circumscribed area of nummular pigmentary deposition with halo of atrophy in inferior peripheral retina. **C**, Nummular pigmentary deposition, yellow-white dots, and clumped pigmentary changes in the mid-peripheral retina. **D**, Yellow-white dots along vascular arcades, with increased fundus autofluorescence inside the vascular arcades, sparing the central macula. **E**, Magnified view of nummular pigmentary deposition, yellow-white dots, and clumped pigmentary changes in mid-peripheral retina. **F**, Torpedo-like lesion in the peripheral retina. **G**, Subretinal fibrosis and spectral-domain OCT tomography across lesion (marked) showing a large subretinal hyperreflective deposit. **H**, Magnified view of yellow-white dots with early pigmentary hyperplastic changes. **I**, Retinal angioma in a patient with bilateral preretinal nondiabetic neovascularization. **J**, Maculopathy, characterized by patchy atrophic macular changes, more visible on fundus autofluorescence. **K**, Color fundus photographs showing the right peripheral retina of patient 11 at baseline (right image) and 17 years later, at last follow-up (left image, year of OCT acquisition marked in left bottom corner). At baseline, retinal sclerosed vessels and yellow-white dots are seen that progressed to nummular and clumped pigmentary deposition as observed in the follow-up photograph of the same area. **L**, Color fundus photographs showing the right superior vascular arcade in patient 15 at baseline (right image) and 11 years later, at last follow-up (left image, year of OCT acquisition marked in left bottom corner). A well-defined area of yellow-white dots is observed at baseline that developed into clumped pigmentary deposition, shown in the follow-up image.

**Figure 3:**
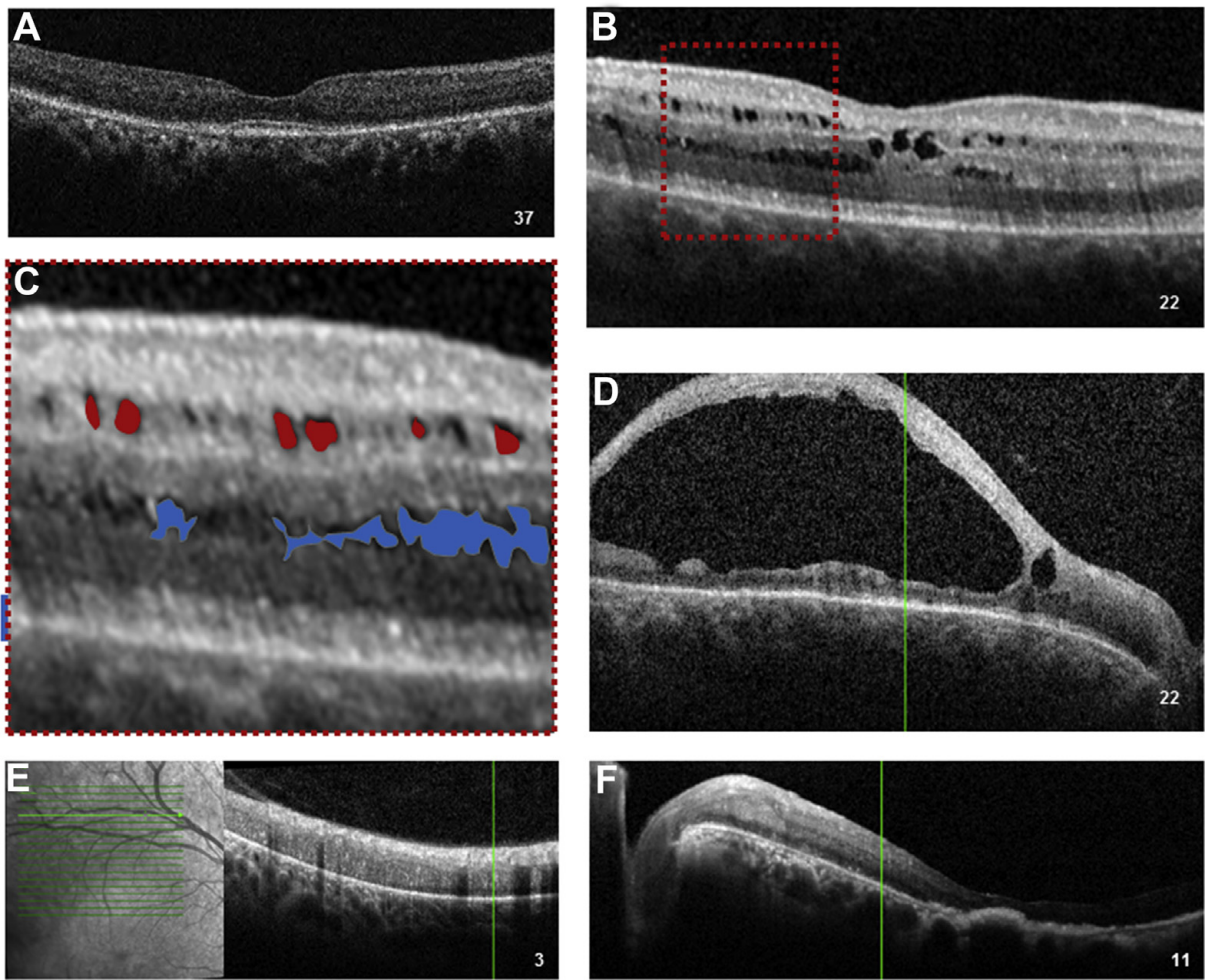
OCT images showing variation of features of enhanced S-cone syndrome in individual patients (numbered). **A**, Preserved foveal architecture and outer retinal atrophy, with loss of the ellipsoid zone. **B**, Foveomacular schisis. **C**, Magnified view of area outlined in (**B**), with pseudocolor representation of the the schitic cavities (round shape, in red) at the level of the inner nuclear layer (round shape, in red) and at the level of the outer nuclear layer (elongated shape, in blue). **D**, End-stage giant foveomacular schisis. **E**, Disorganization of retinal layers in the atrophic area of mid-peripheral retina. **F**, Macular atrophy.

**Figure 4:**
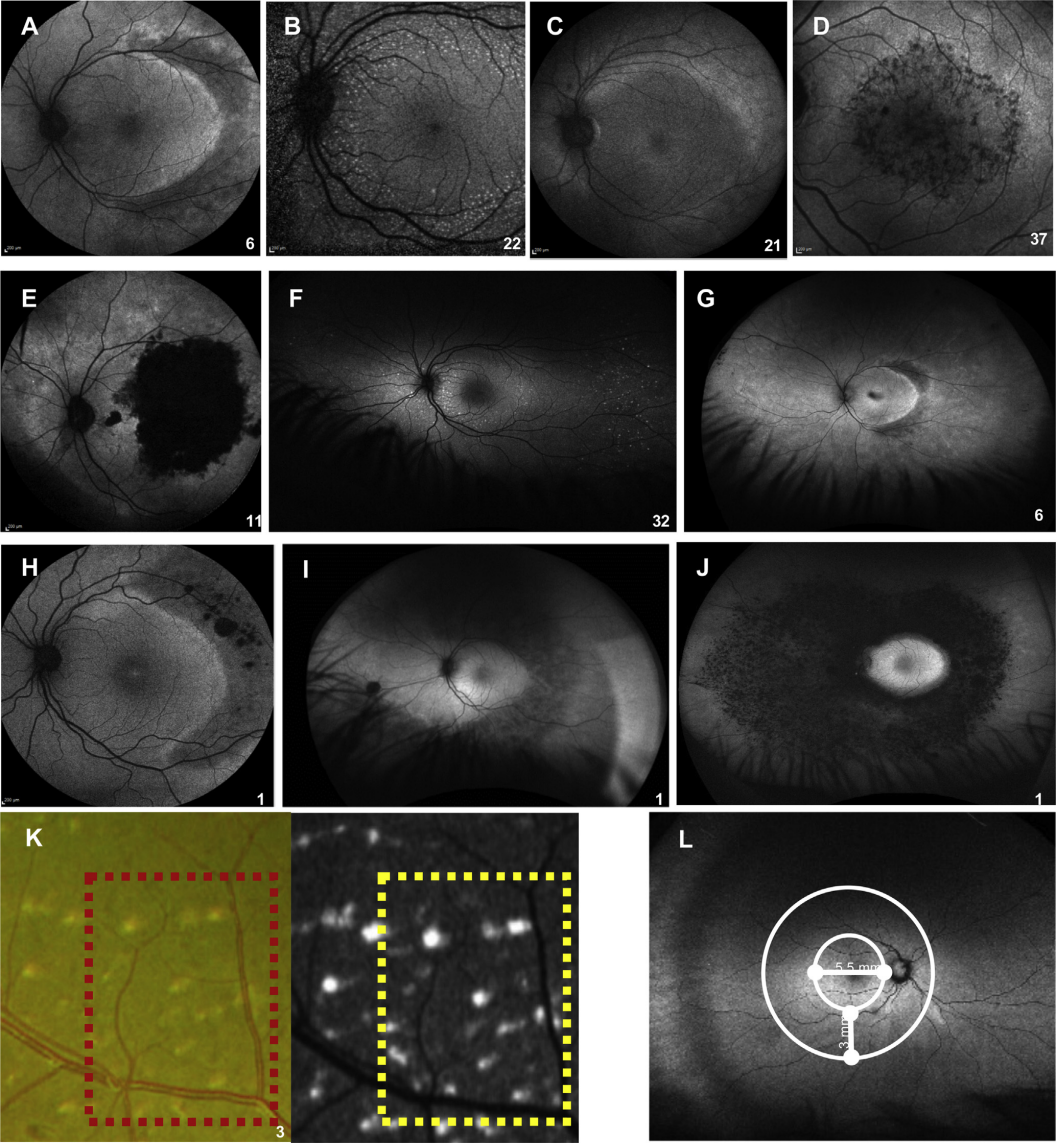
Fundus autofluorescence (FAF) images showing macular and peripheral patterns in enhanced S-cone syndrome in individual patients (numbered). **A**, Minimal change macular FAF pattern. **B**, Minimal change macular FAF pattern with hyperautofluorescent flecks. **C**, Mild diffuse macular hypoautofluorescence. **D**, Moderate speckled macular hypoautofluorescence with increased paramacular FAF. **E**, Severe end-stage macular hypoautofluorescence. **F**, Peripheral hyperautofluorescent flecks. **G**, Moderate diffuse (mid-peripheral half-ring or ring <5000 μm widest diameter) peripheral hypoautofluorescence with half-ring of pronounced hyperautofluorescent ring along the temporal macular rim. **H**, Near-peripheral moderate diffuse hypoautofluorescence with patchy advanced hypoautofluorescence. **I**, Moderate diffuse peripheral hypoautofluorescence (>5000 μm). **J**, Advanced peripheral hypoautofluorescence. **K**, Colour fundus photograph and related autofluorescence image showing the correspondence between yellow-white dots and hyperautofluorescent flecks. **L**, Wide-field autofluorescence image from a control participant. The macula was defined as the region encompassing 5.5 mm from the temporal margin of the optic nerve head and the mid periphery as 3 mm around the macula.

Clinical notes, imaging data, and color fundus photographs were reviewed in all patients. Color fundus photographs and OCT scans of a representative group of patients depicting the below-mentioned clinical features are in Figures 2 and 3. In most cases, yellow-white dots, nummular pigmentation, or both were observed, located within the vascular arcades in 16 patients (28.6%) and in the mid-peripheral retina, outside the vascular arcades, in 49 patients (87.5%; Fig 2A–E). Nummular pigmentation, characterized by deep round pigmentation at the level of the RPE, was usually located in the mid-peripheral retina, along the vascular arcades, and often associated with RPE atrophy and end-stage hypoautofluorescence (Fig 4J). This was the most common clinical finding, present in 48 patients (85.7%), followed by yellow-white dots, which were seen in 32 patients (57.1%). The combined presence of nummular pigmentation with yellow dots was observed in 26 patients (46.4%). Two patients demonstrated clumped and nummular pigmentary changes in an area of the mid-peripheral retina with yellow-white dots (Fig 2K, L).

Other clinical findings included vitreous opacities (21.4%), peripheral torpedo-like lesions (10.7%; Fig 2F), circumferential subretinal fibrosis (7.1%; Fig 2G), optic disc pallor (5.4%), and recurring vitreous hemorrhages secondary to preretinal neovascularization in 1 patient (1.8%; Fig 2I).

Foveomacular schisis was identified in 23 of 56 patients (41%; Fig 3B, C). Two patients demonstrated giant foveomacular schisis (Fig 3D) that evolved into advanced macular atrophy in 1 eye (Figs 3F and 4E). In all other patients, OCT appearances were relatively stable. The schitic cavities were located at the level of the inner nuclear layer, characterized by a round shape, and in the outer nuclear layer, where they appeared elongated in a stellate-like configuration (Fig 3C). A total of 16 patients (28.6%) were diagnosed with presumed cystoid macular edema (CME) based on structural appearance on spectral-domain OCT. Four patients were treated with oral carbonic anhydrase inhibitors. Resolution of CME was achieved in 2 patients, albeit with no visual gain, including in the single patient in whom, before treatment, leakage had been demonstrated on fluorescein angiography.

Most patients (55%) demonstrated minimal autofluorescent changes at the level of the macula (minimal change pattern), combined with the presence of hyperautofluorescent flecks in some cases. End-stage macular atrophy with secondary severe macular hypoautofluorescence was observed in 1 patient, and this was preceded by giant foveomacular schisis. The hyperautofluorescent flecks correlated with the presence of yellow-white dots (Fig 4K, L). In the peripheral retina, a moderate decrease in autofluorescence was observed in most patients (31.7%), usually combined with patchy severe hypoautofluorescence, the latter corresponding to the presence of nummular pigmentary deposition. A strong half-ring of pronounced hyperautofluorescence along the temporal macular rim was observed in all patients demonstrating peripheral half-ring hypoautofluorescence. In 5 patients, the peripheral FAF pattern progressed from a moderate decrease in autofluorescence to a patchy decrease in autofluorescence, with documented progression of pigmentary changes. Clinical FAF patterns are shown in Figure 4 and summarized in Supplemental Table 1 (available at www.ophthalmologyretina.org), where the mean BCVA is presented in relation to the pattern of macular FAF findings.

### Electrophysiologic Findings

**Figure 5:**
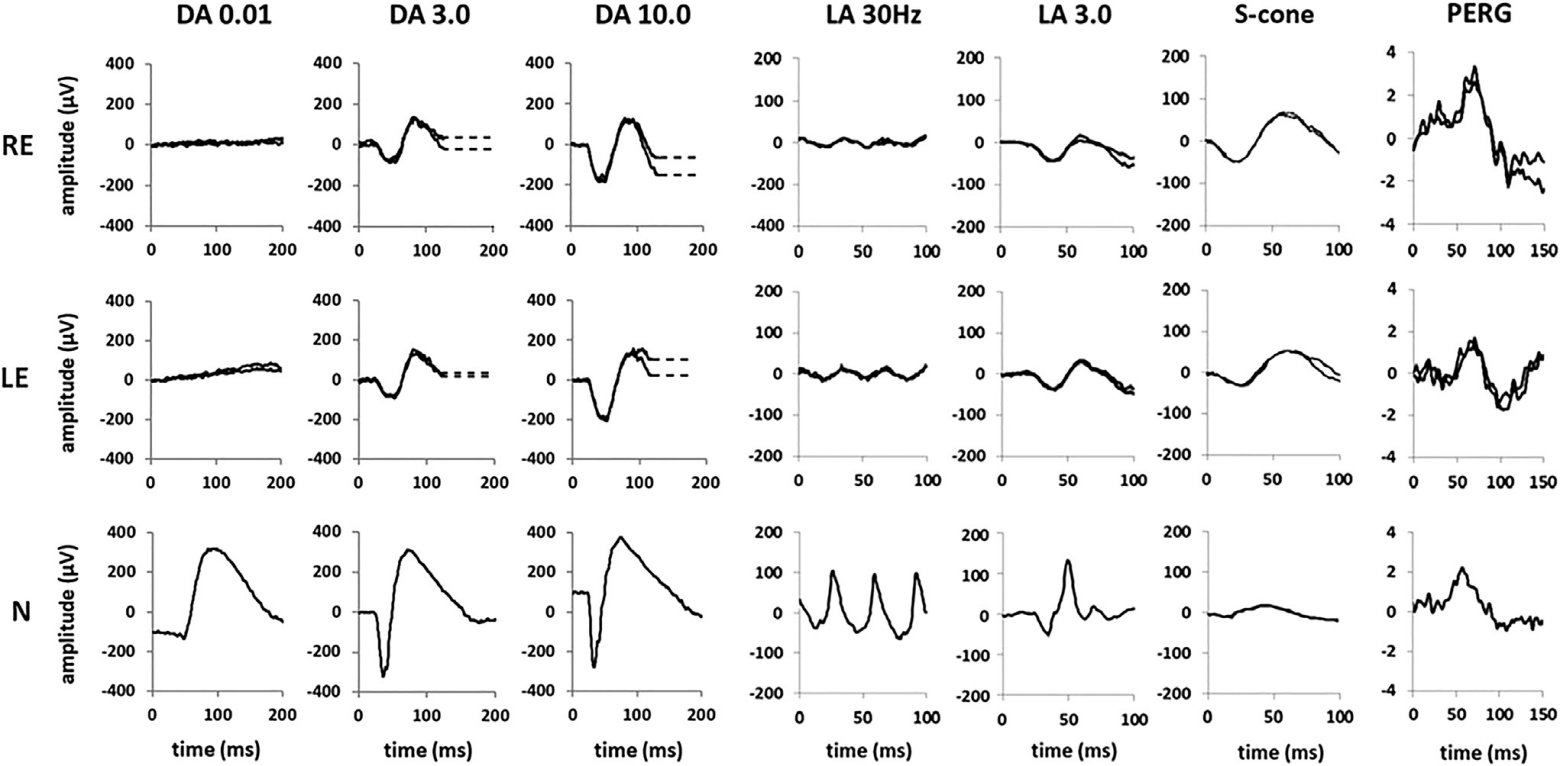
Full-field electroretinography and pattern electroretinography (PERG) recordings from the right eye (RE) and left eye (LE) of a patient with enhanced S-cone syndrome compared with recordings from a representative unaffected control participant (N). Electroretinography recordings include the dark-adapted (DA) electroretinography responses (flash strengths, 0.01 and 10.0 cd.s/m^2^; DA 0.01 and DA 10.0) and light-adapted (LA) electroretinography responses for a flash strength of 3.0 cd.s/m^2^ (LA 3.0; 30 Hz and 2 Hz). The pattern electroretinography (PERG) responses are recorded in an alternating checkerboard. A 20-ms prestimulus delay in single-flash electroretinography recordings is present, with the exception of the S-cone electroretinography response. Broken lines replace blink artefacts occurring after electroretinography b-waves, for clarity. Patient responses are superimposed to demonstrate reproducibility. In this patient, the PERG P50 component is delayed but of normal amplitude. The rod-specific dim flash (DA 0.01) electroretinography features are undetectable. The single-flash DA 3.0, DA 10.0, and LA 3.0 electroretinography responses have similarly simplified and severely delayed waveforms, qualitatively comparable in shape with the S-cone electroretinography response and consistent with generation by the same (S-cone) mechanism. The S-cone electroretinography response is delayed and grossly enlarged. The LA 30-Hz electroretinography response is smaller than the LA 3 electroretinography a-wave, whereas in the typical healthy participant, the LA 30-Hz electroretinography amplitude is between that of the LA 3 a- and b-waves. Measurements of the main electroretinography components are compared with the control range in Supplemental Table O (available at www.ophthalmologyretina.org).

The PERG P50 component (Supplemental Fig 1, available at www.ophthalmologyretina.org) was undetectable (n = 11), delayed and reduced (n = 11), delayed and of normal amplitude (n = 3; see Fig 5 for an example), or normal (n = 3). The threshold values for the PERG P50 minimum amplitude and maximum peak time are presented in Supplemental Table 2 (available at www.ophthalmologyretina.org). No correlation was found between the PERG P50 amplitude and visual acuity in right eyes (*r* = –0.20; *P* = 0.277; n = 31) or left eyes (*r* = –0.18; *P* = 0.326; n = 31). Significant negative correlation was found between age and PERG P50 amplitude for right eyes (*r* = 0.65; *P* < 0.05; n = 36) and left eyes (*r* = 0.6; *P* < 0.05), and positive correlation was found between age and P50 peak time for right eyes (*r* = 0.6; *P* < 0.05; n = 23) and left eyes (*r* = 0.55; *P* < 0.05).

**Figure 6:**
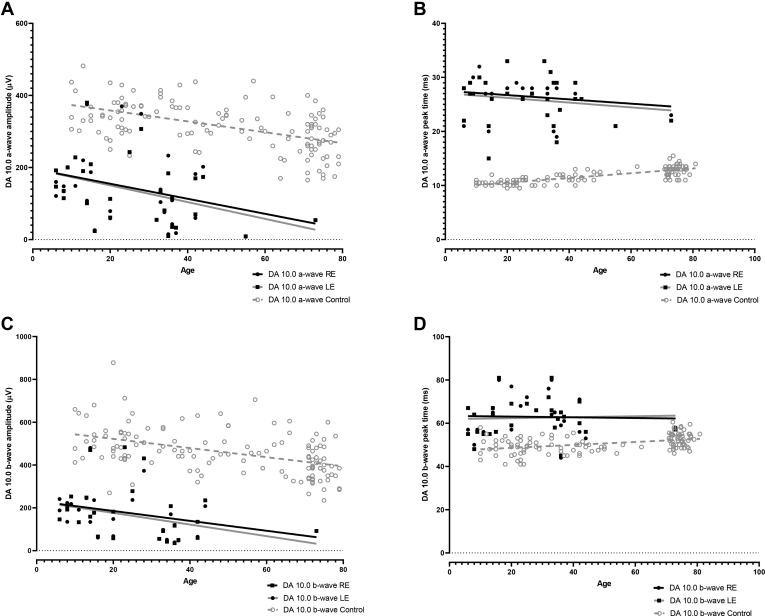
Graphs showing the main dark-adapted (DA) full-field electroretinography component amplitudes and peak times in each eye in the enhanced S-cone syndrome (ESCS) cohort (filled circles and squares) and in healthy control participants (gray circles) plotted against age (in years) at the time of testing, illustrating the severity and range of electroretinography abnormality in the ESCS group. Data are shown for the DA strong flash (DA 10) electroretinography a-wave (**A**) amplitude and (**B**) peak time and for the b-wave (**C**) amplitude and (**D**) peak time. Regression analysis shows a similar, statistically significant (*P* < 0.05) age-related reduction in amplitudes for both control (broken lines) and ESCS (solid lines) groups. LE = left eye; RE = right eye.

**Figure 7:**
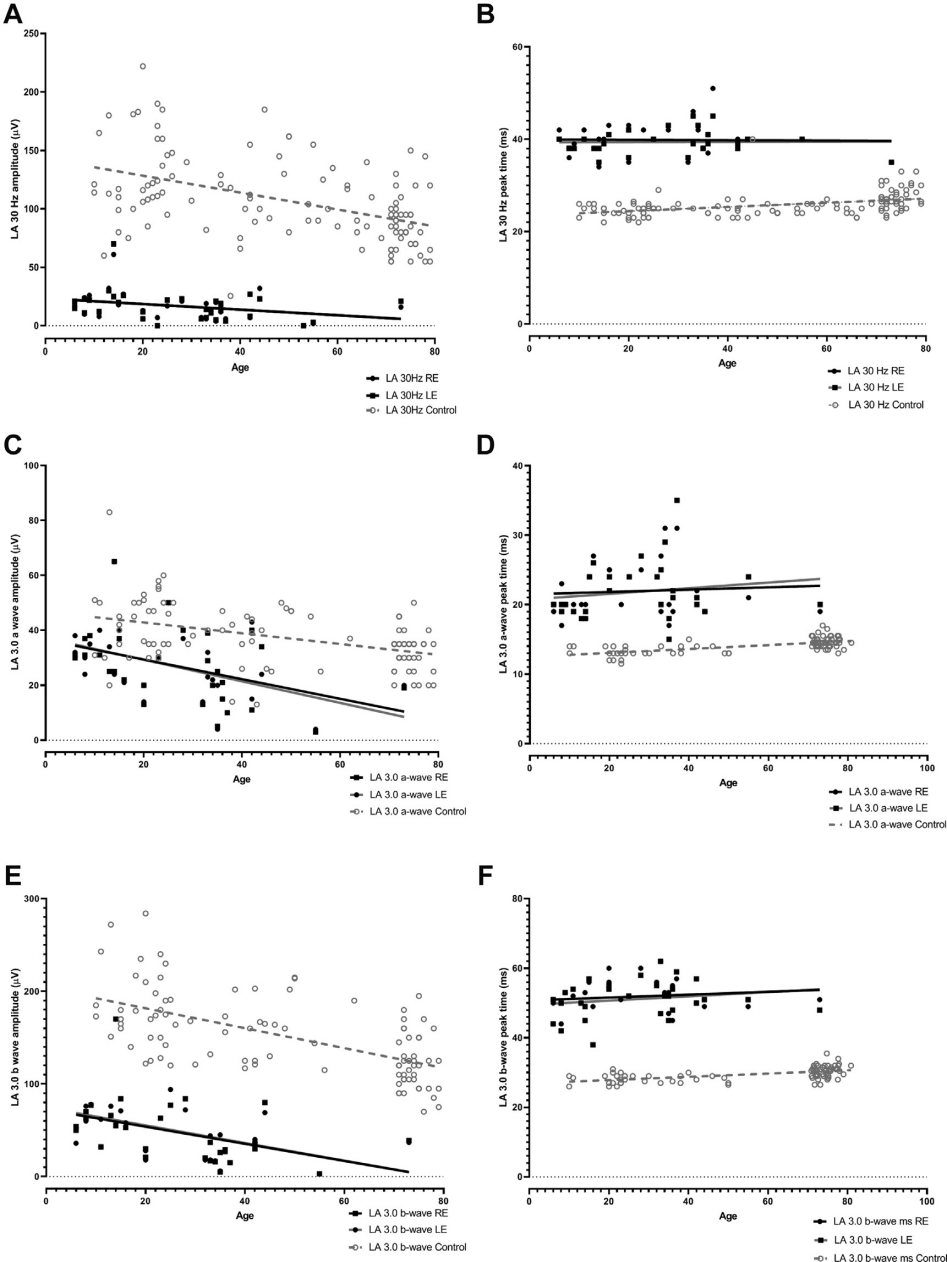
Graphs showing the main light-adapted (LA) full-field electroretinography component amplitudes and peak times in each eye in the enhanced S-cone syndrome (ESCS) cohort (filled circles and squares) and in healthy control participants (gray circles) plotted against age (in years) at the time of testing, illustrating the severity and range of electroretinography abnormality in the ESCS group. Data are shown for the LA 30-Hz flicker electroretinography (**A**) amplitude and (**B**) peak time, for the single flash cone (LA 3) electroretinography a-wave (**C**) amplitude and (**D**) peak time, and for the LA 3 electroretinography b-wave (**E**) amplitude and (**F**) peak time. Regression analysis shows a similar, statistically significant (*P* < 0.05) age-related reduction in amplitudes for both control (broken lines) and ESCS (solid lines) groups. LE = left eye; RE = right eye.

The full-field electroretinography waveforms were undetectable in 1 patient with a genetically confirmed diagnosis. All other individuals who underwent ISCEV-standard testing (n = 31) showed pathognomonic electroretinography abnormalities, as described below (typical recordings are shown in Fig 5). The DA 3.0, DA 10.0, and LA 3.0 electroretinography responses showed a similar simplified and delayed waveform shape. The ranges of full-field electroretinography component amplitudes and peak times are compared with those in a control group in Figures 6 and 7. The DA rod-specific dim flash (DA 0.01) electroretinography features were undetectable in all but 1 patient (age, 14 years) with a detectable but subnormal response (reduction of 58% compared with the mean for the control group). The DA 10.0 electroretinography a- and b-wave mean amplitudes were reduced by 52% and 63%, respectively, and mean a- and b-wave peak times were 16 ms and 14 ms longer, respectively, compared with those for the control group (Fig 6).

**Figure 8:**
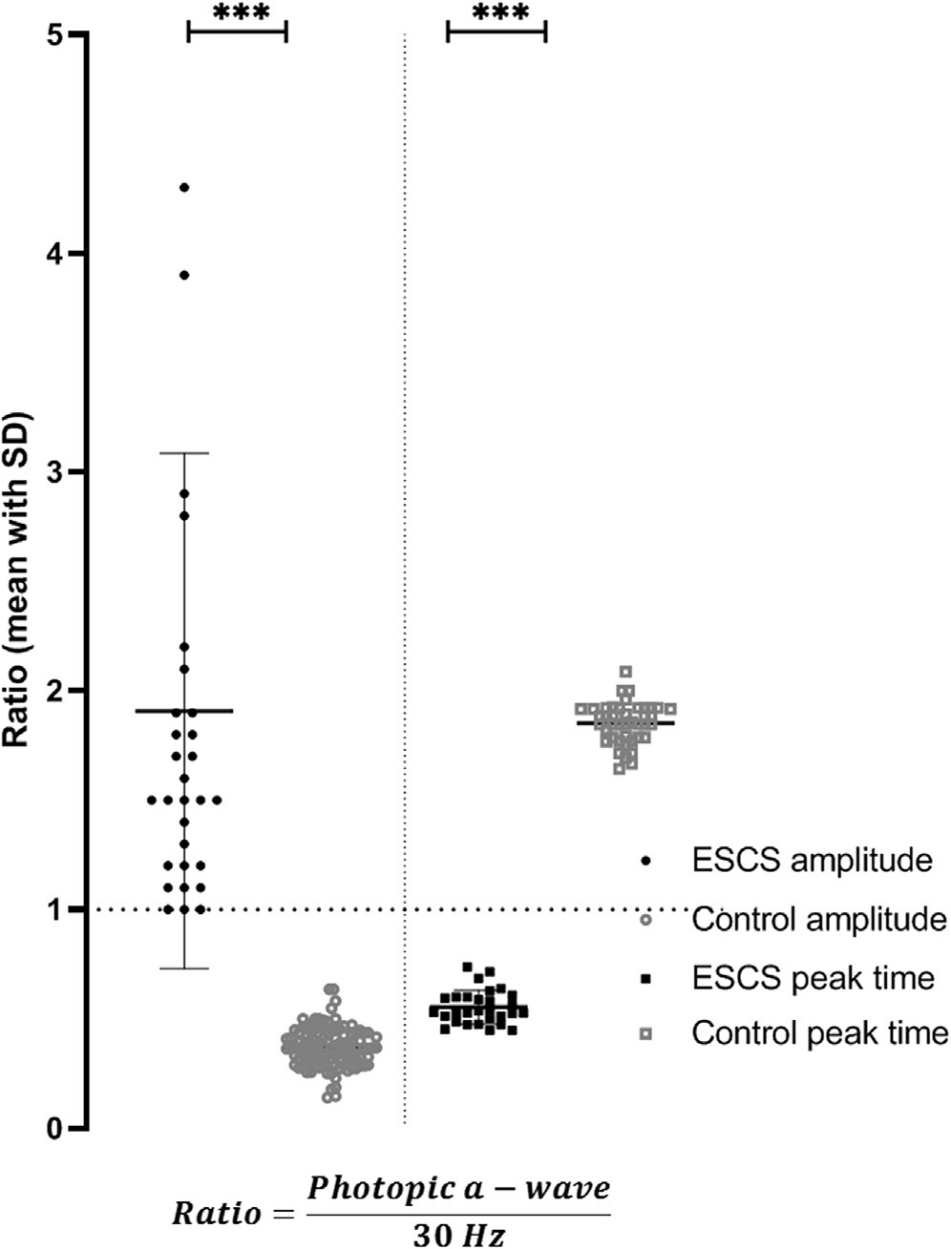
Graph showing a comparison of amplitude and peak time ratios between the light-adapted (LA) 3.0 electroretinography a-wave and LA 30-Hz electroretinography responses in the enhanced S-cone syndrome (ESCS) cohort (filled circles and squares) and healthy control participants (open gray circles and squares). The horizontal bars show the mean ±1 standard deviation (SD) for amplitudes in the ESCS group. The LA 30-Hz electroretinography response has an amplitude of more than the LA 3 electroretinography a-wave in all control participants. In the ESCS group, the LA 30-Hz electroretinography amplitude is equal or smaller than the LA 3 electroretinography a-wave, resulting in a ratio of 1 or more. ∗∗∗*P* = 0.0001.

The LA 30-Hz electroretinography and LA 3.0 electroretinography a- and b-wave amplitudes were, on average, 93%, 28%, and 69% lower, respectively, and mean peak times were 14 ms, 8 ms, and 20 ms greater, respectively, compared with mean values for the control group (Fig 7). The LA 30-Hz flicker electroretinography response was smaller than the LA 3.0 electroretinography a-wave in most participants (n = 48 eyes of 27 participants) and of equal amplitude to the LA 3.0 electroretinography a-wave in others (n = 9 eyes of 7 participants), including the 4 eyes with the smallest detectable responses. The mean amplitude ratio between the LA 3.0 electroretinography a-wave and LA 30-Hz electroretinography response was 1.86 (n = 30; 44.53% coefficient of variation; SD, 0.9; Fig 8) in the ESCS cohort and 0.37 (n = 111; 24.38% coefficient of variation; SD, 0.09) in the healthy control participants. The peak time ratio between the LA 3.0 electroretinography a-wave and LA 30-Hz electroretinography responses was 0.55 (n = 30; 14.02% coefficient of variation; SD, 0.08; Fig 8) in the ESCS cohort (*P* = 0.0001) and 1.85 (n = 42; 5.0% coefficient of variation; SD, 0.09) in the healthy control participants.

**Figure 9:**
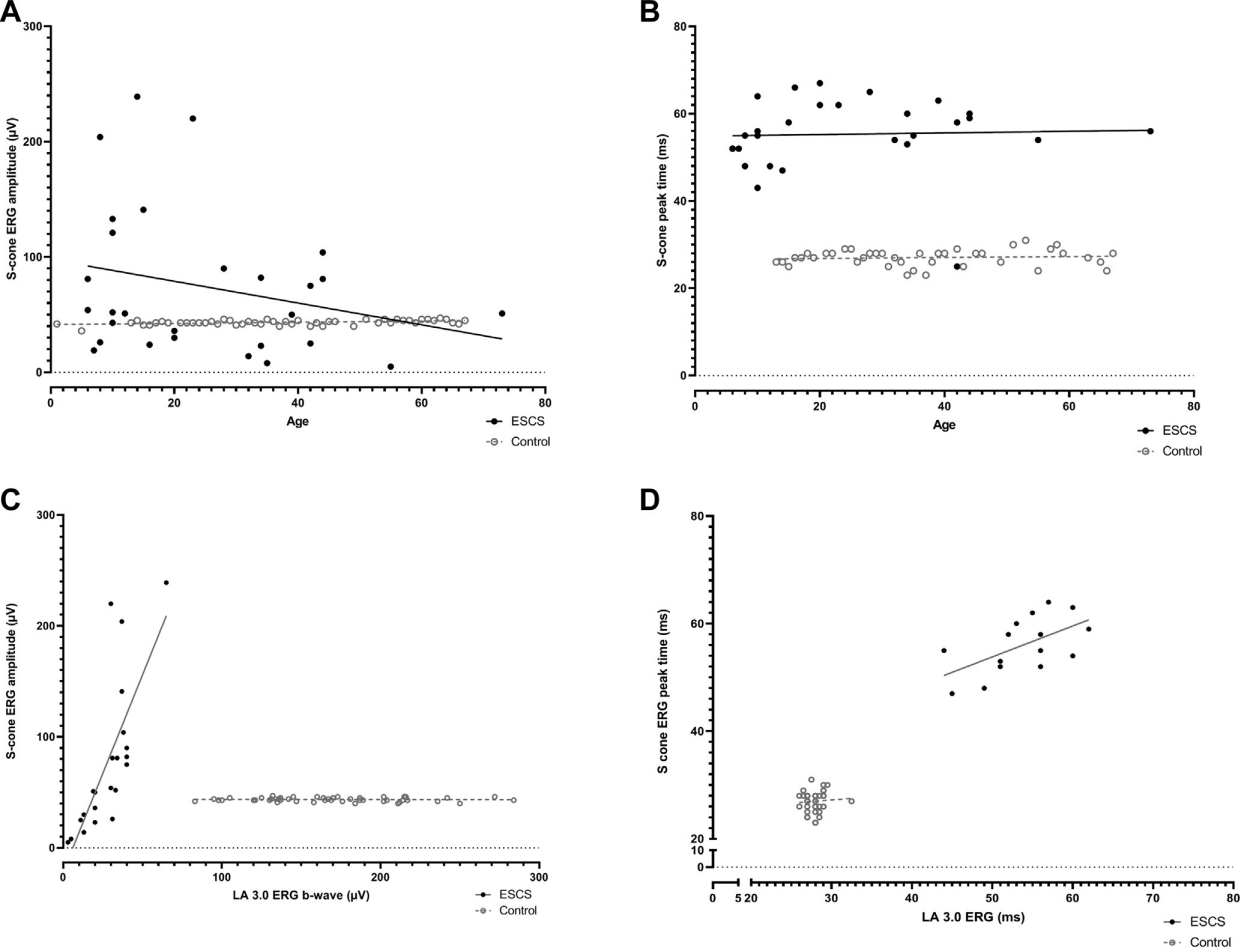
Graphs showing the S-cone electroretinography (ERG) component (**A**) amplitudes and (**B**) peak times for the enhanced S-cone syndrome (ESCS) cohort (n = 28, filled circles) and a healthy control group (n = 51, open gray circles) for comparison, plotted against age (in years). S-cone electroretinography amplitudes were measured from the early negative trough to maximum peak or, in the absence of an early trough, from baseline to the peak of the positive polarity S-cone electroretinography component. The largest S-cone electroretinography responses were seen in some of the younger ESCS individuals (solid regression line shows a negative slope), but no age-related statistically significant differences were found. Comparison of S-cone electroretinography (**C**) amplitudes and (**D**) peak times with those for light-adapted (LA) 3 electroretinography b-waves are shown for the ESCS and control groups and illustrate high positive correlation in the ESCS group, consistent with S-cone and LA 3 electroretinography responses being dominated by abnormal S-cone opsin-mediated activity. All data relate to right-eye recordings.

The mean S-cone electroretinography amplitude in the ESCS patients was greater (mean, 81 μV; median, 54 μV; n = 28; mean age, 27 years) than in the control group (mean, 43.35 μV; median, 43 μV; n = 51; mean age, 29 years; Fig 9A), and peak times were severely delayed (mean peak time difference [ESCS – control] = 28.3 ms; Fig 9B). S-cone electroretinography responses in ESCS patients were largest in some of the children and young adults, but no significant correlation was found between amplitude or peak time and age (*r*^2^ = 0.06 and *r*^2^ = 0.001, respectively; *P* > 0.05; Fig 9A, B). In ESCS patients, significant correlation was found between the S-cone electroretinography and LA 3 electroretinography b-wave amplitudes (*r*^2^ = 0.56; *P* < 0.001) and peak times (*r*^2^ = 0.34; *P* < 0.05; Fig 9C, D).

Plots of the major ISCEV-standard electroretinography component amplitudes and peak times against age are shown in Figures 6 and 7. Evidence of age-related electroretinography reduction was found in the DA and LA electroretinography responses at a rate that was indistinguishable from that seen in healthy participants over 6.7 decades (LA 3.0 electroretinography a-wave, *r*^2^ = 0.22 [*P* = 0.006]; LA 3.0 electroretinography b-wave, *r*^2^ = 0.22 [*P* = 0.007]; DA 10 a-wave, *r*^2^ = 0.17 [*P* = 0.02]; and DA 10 b-wave, *r*^2^ = 0.16 [*P* = 0.03]). The peak times of the major electroretinography components in ESCS patients showed high stability with increasing age, as in the control group.

The patient with compound heterozygous changes in *NR2E3* (c.119-2A→C and the novel p.L303P) had a particularly severe clinical phenotype with early onset of visual symptoms, severely reduced visual acuity (1.0 logMAR), sensory nystagmus, and giant foveomacular schisis that evolved into end-stage macular atrophy (Supplemental Fig 2, available at www.ophthalmologyretina.org). His electroretinography results, obtained when he was 53 years of age, differed from those of all other patients, characterized by undetectable DA and LA electroretinography responses and an undetectable PERG response.

Five individuals underwent follow-up electroretinography testing after intervals of 4, 6, 9, 10, and 17 years. The mean annual rate of electroretinography reduction (averaged between eyes) for DA 10 electroretinography a- and b-wave amplitudes was 1.6% (range, 0%–6.0%) and 3.9% (range, 0%–6.2%), respectively; for LA 3 electroretinography a- and b-wave amplitudes, the mean rate of reduction was 3.4% (range, 2.0%–4.7%) and 1.3% (range, 0%–2.0%), respectively.

### Molecular Genetics

Forty-one of 56 patients underwent screening of the 9 coding exons of *NR2E3*. Disease-causing variants were identified in 41 participants. Twenty-four participants were homozygous and 17 showed compound heterozygous variants. Eighteen sequence variants were identified, including 4 novel missense variants (p.F71L, p.R247W, p.L303P, and p.R309Q; Table 4). The other reported variants encompassed 2 splice acceptor variants in intron 1 (c.119-2A→C^22^ and c.119-3C→G^33^), 10 missense mutations (p.R76Q,^22^ p.C83Y,^60^ p.A102D,^44^ p.R104W,^22^ p.G216S, p.R104Q,^27^ p.R311Q,^16^ p.A256E,^5^ p.V342A,^44^ and p.L371W^61^), 1 frameshift mutation (p.P399Qfs∗3^44^), and a 9-bp deletion leading to deletion of 3 amino acid residues (p.C67_G69del^16^).

**Table 4:** *NR2E3* Variants

Exon	Nucleotide Substitution and Amino Acid Change	Previous Report (Reference No.)	Clinical Interpretation of Genetic Variants∗	Protein Variation Effect Analyzer†		PolyPhen 2‡		Allelic Frequency
			Prediction	Prediction	Index	Prediction	Human Variation Score (0–1)	
IVS1	c.119-2A→C	22			NA		NA	0.0005031
IVS1	c.119-3C→G	33			NA		NA	Not reported on gnomAD
2	c.211T→C; p.F71L	Novel	Uncertain significance	Deleterious	–5.218	PRD	1.00	Not reported on gnomAD
2	c.200_208del9del; p.C67_G69del	22		Deleterious	–26.485		NA	0.00001347
2	c.227G→A; p.R76Q	22	Uncertain significance	Deleterious	–3.343	PRD	1.00	0.0002140
3	c.248G→A; p.C83Y	83	Uncertain significance	Deleterious	–9.185	PRD	1.00	0.00001308
3	c.305C→A; p.A102D	44	Likely pathogenic	Deleterious	–4.862	PRD	0.99	0.00002792
3	c.310C→T; p.R104W	22	Uncertain significance	Deleterious	–6.962	PRD	1.00	0.00001964
5	c.646G→A; p.G216S	22	Uncertain significance	Neutral	0.491	Benign	0	0.00003611
5	c.311G→A; p.R104Q	27	Likely pathogenic	Deleterious	–3.533	PRD	1.00	0.00001964
5	c.739C→T; p.R247W	Novel	Uncertain significance	Deleterious	–7.733	PRD	1.00	Not reported on gnomAD
6	c.908T→C; p.L303P	Novel	Uncertain significance	Deleterious	–6.552	PRD	1.00	Not reported on gnomAD
6	c.932G→A; p.R311Q	22	Likely pathogenic	Neutral	–1.831	PSD	0.627	0.0004071
6	c.767C→A; p.A256E	84	Likely pathogenic	Deleterious	–3.659	PRD	0.998	0.00003715
6	c.926G→A; p.R309Q	Novel	Likely pathogenic	Deleterious	–3.520	PRD	0.959	0.00001528
7	c.1025T→C; p.V342A	85	Uncertain significance	Deleterious	–3.677	PRD	0.996	Not reported on gnomAD
8	c.1194delT; p.P399Qfs∗3	85			NA		NA	Not reported on gnomAD
8	c.1112T→G; p.L371W	86	Uncertain significance	Deleterious	–5.107	PRD	1.00	Not reported on gnomAD

gnomAD = Genome Aggregation Database; NA = not applicable; PRD = probably damaging; PSD = possibly damaging.

∗ According to the 2015 American College of Medical Genetics and Genomics and the Association for Molecular Pathology guidelines, which categorize causality of variants as pathogenic, likely pathogenic, uncertain significance, likely benign, and benign (http:// http://wintervar.wglab.org; accessed 12.06.20).

† http://provean.jcvi.org/index.php; accessed 15.02.19. Variants with a of –2.5 or less are considered deleterious. Variants with a score of more than –2.5 are considered neutral.

‡ Polyphen 2 (vision 2.1) appraises mutations qualitatively as benign, possibly damaging, or probably damaging based on the model's false positive rate (http://genetics.bwh.harvard.edu/pph2/; accessed 28.02.20). HumanVar-trained model of Polyphen 2 was selected because the diagnostics of Mendelian diseases require distinguishing mutations with drastic effects from all the remaining human variations, including abundant mildly deleterious alleles.

The p.G216S substitution (c.646G→A; exon 5) was found as a homozygous change in 1 patient. This variant, predicted to be benign, is rare in gnomAD, but the amino acid change is not predicted to be damaging by any of the in silico tools used. However, this may be irrelevant to causality. The variant introduces an exonic splice acceptor site: TGCGGCC→tgcagCC (human splice finder score, 91.23; nnsplice score, 0.97) into exon 5 that is likely to lead to an out-of-frame deletion of the 5′ 77 bp of exon 5 and may thus represent a loss-of-function allele. Without mRNA analysis of relevant patient tissue, it is not possible to determine if this predicted splice-altering effect is indeed occurring in vivo or if any normally spliced transcript would escape and produce functional protein, but we are of the opinion that the case for causality is sufficient for this rare variant.

**Figure 10:**
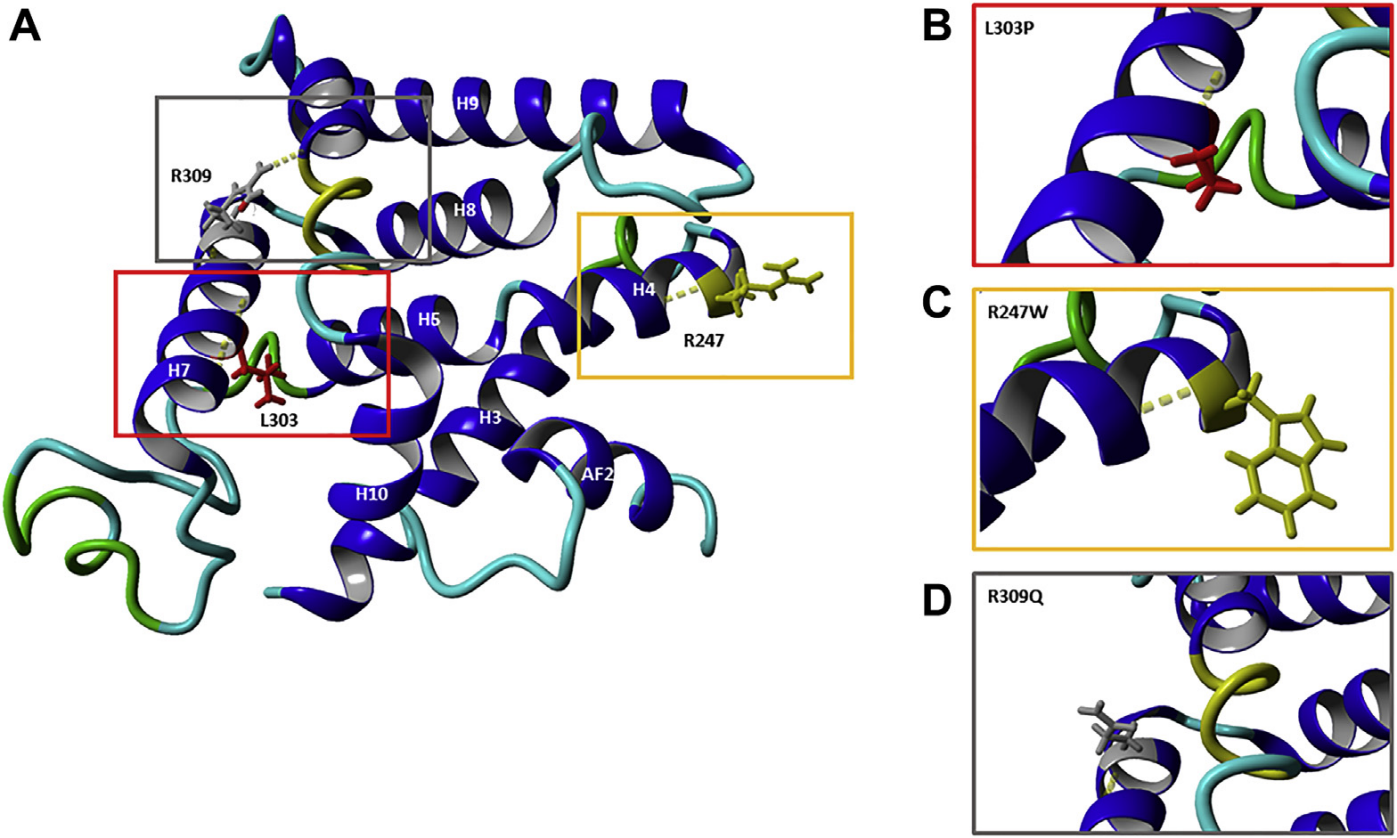
**A**, Stereo representation of the *NR2E3* ligand-binding domain (LBD) monomer (pdb code, 4LOG), starting at residue 217, and locations of the novel missense *NR2E3* LBD mutations mapped on the receptor. **B**–**D**, Predicted effect of the mutations (p.R247W, p.L303P, and p.R309Q) leading to a rearrangement of the bulky side chains and loss of hydrogen bonds.

Four novel disease-causing variants were identified. These are likely to be pathogenic, given that all are located within highly conserved domains critical to protein function and all are rare or absent from control datasets. The p.F71L substitution (c.211T→C; exon 2) was found as a heterozygous change in 1 patient. The p.R247W substitution (c.739C→T; exon 5) was found as a heterozygous change in 1 patient. The p.L303P substitution (c.908T→C; exon 5) was found as a heterozygous change in 1 patient. The p.R309Q substitution (c.926G→A; exon 6) was found in a homozygous state in 2 affected siblings. The Phe71, Arg247, Leu309, and Arg309 are highly conserved across *NR2E3* orthologs. *NR2E3* has the evolutionarily conserved modular structure of nuclear receptors, namely a highly conserved DNA-binding domain that specifically binds to consensus binding sites located in promoters of target genes, and a ligand-binding domain.^12^^,^^13^^,^^62^ Three of the aforementioned novel mutations, p.R247W, p.L303P, and p.R309Q, are located in the evolutionary-conserved ligand binding domain of *NR2E3*, in helices 4 and 7, causing a rearrangement of the bulky side chains and loss of some hydrogen bonds, suggesting a reduction of protein stability (Fig 10). The p.F71L is located in the evolutionary-conserved DNA binding domain of *NR2E3*.^59^^,^^63^ Definite confirmation of the pathogenicity of the 4 novel variants remains dependent on functional studies that would assess the effects of these sequence variants with regard to *NR2E3* stability, targeting, and ability to interact reversibly and effectively with DNA or ligands.

## Discussion

This study described the largest cohort of patients diagnosed with ESCS. We characterized the clinical variability and described molecular characteristics, including 4 novel variants in *NR2E3*. Detailed quantification of the electrophysiologic findings characterizes the phenotypic variability of pathognomonic electroretinography features and assesses the relative stability of macular and retinal dysfunction over 6 decades, pertinent to possible future interventional studies.

Enhanced S-cone syndrome is characterized by early onset of visual symptoms. In this cohort, all but 2 patients experienced symptoms in the first 2 decades of life, with most seeking treatment in childhood. Nyctalopia, with or without reduced central visual acuity, was the most frequently described initial symptom. Hyperopia with a variable degree of astigmatism was the most common refractive error, in accordance with other reports.^5^^,^^33^^,^^64^

Sparing along the tritan axis was demonstrated in 6 patients who underwent color contrast sensitivity testing, suggesting preservation of short-wavelength discrimination. This is also consistent with the high amplitude S-cone electroretinography responses seen in many individuals and with high correlation between S-cone electroretinography responses and LA 3 electroretinography responses, likely having identical S-cone opsin-mediated origins.

Visual function was highly variable among patients, ranging from normal to severely reduced (2.0 logMAR). It is noteworthy that in most patients, BCVA remained relatively stable throughout follow-up, with no clinical progression observed in 79 of 100 eyes. The slight deterioration in BCVA with increasing age may be ascribed to the expected age-related decline in the general population. In 2 patients, poor visual outcome was related to nondystrophic significant ophthalmic events (retinal detachment and dense amblyopia). Poorer visual outcomes were associated with the presence of moderate to advanced (giant) foveomacular schisis, but no other association was found, either with age at onset of visual symptoms or with genotype or electrophysiologic responses. Also, a high degree of interocular symmetry was found that could enable the use of the contralateral eye as a valid untreated control in future therapeutic trials in which 1 eye received treatment.

In the family demonstrating a pseudodominance pattern, clinical severity was highly variable. Whereas the father was found to have severely reduced BCVA in both eyes when first assessed at 17 years of age, his children showed mildly reduced BCVA when tested at an equivalent age. Interestingly, clinical presentation was not only variable within the same family but also observed in patients from different families harboring the same variant, suggesting that modifier genes (and environmental factors) may modulate disease outcome.^26^^,^^65^

One patient demonstrated bilateral nondiabetic preretinal neovascularization and a midperipheral vasoproliferative lesion in 1 eye that led to recurrent vitreous hemorrhages (Fig 2I). This is an unusual finding, and whether this is related to the underlying retinal dystrophy remains unanswered. However, choroidal neovascularization was described previously in patients with ESCS. Asymptomatic development of choroidal neovascularization has also been linked to the presence of torpedo-like lesions and circumferential subretinal fibrosis, both infrequent findings in ESCS.^40^^,^^66^, ^67^, ^68^

Clinical appearance was highly variable; however, 3 consistent clinical signs were observed in a large proportion of patients: yellow-white dots, nummular pigmentation at level of the RPE, and foveomacular schisis. In the appropriate clinical context, the presence of these combined features should raise the strong possibility of ESCS.

The yellow-white dots are often characterized by an increase in autofluorescence signal and present in both the macula and midperiphery at the level of the RPE. Histologic analysis of autofluorescent white dots seen across the retina of the rd7 mouse, which harbors a homozygous deletion in *NR2E3*, showed that the autofluorescence signal arose mostly from macrophages, which were associated with whorls and rosettes of dysplastic photoreceptors in the outer nuclear layer.^69^ Further in vivo studies are warranted to ascertain the exact origin of the hyperautofluorescent dots observed in ESCS patients.

Nummular pigmentary deposition alone is not specific to ESCS and has been described in other retinal dystrophies such as Bardet-Biedl syndrome,^70^ *CRB1*-associated early-onset severe retinal dystrophy,^71^ retinitis pigmentosa with preserved para-arteriolar RPE (RP12, associated with *CRB1*),^72^ and thioridazine retinopathy.^73^ Whenever present, nummular pigmentary deposition was associated with disorganization of the neurosensory retina, including marked loss of the ellipsoid zone and absence of autofluorescence, in keeping with previous reports.^23^^,^^33^^,^^35^ In some patients with mid-peripheral, nummular pigmentation, clumped pigmentary deposition was observed. The presence of yellow-white dots has been proposed as a harbinger of more marked pigmentary changes, developing early in the pathogenesis of the disease, followed by the development of nummular and clumped pigmentary deposition at a later stage.^5^ Corroborating this assumption, documented progression of pigmentary changes over time was observed in 2 patients with extended follow-up. The development of pigmentary changes occured independent of age. In our cohort, clumped or nummular pigmentary deposition, although skewed toward older participants, was present in 11 young patients (age, ≤20 years) and absent in 4 older patients (age, >20 years), corroborating the high variability of clinical phenotype.

The scarcity of fundus fluorescein angiography in the diagnosis of CME poses an important limitation, because we are unable to confirm CME solely based on spectral-domain OCT structural appearance. It is possible that the presumed CME documented in many patients represents a variant of foveomacular schisis that mimics the appearance of cystoid spaces. A positive anatomic response to carbonic anhydrase inhibitors was observed in only 2 patients, although this did not translate into a significant gain in subjective and objective visual function. Notwithstanding, poorer visual outcomes were associated with macular changes, namely foveomacular schisis, presumed CME, and macular atrophy, rendering prevention and treatment of maculopathy an invaluable target in future treatment strategies.

Pattern electroretinographys ranged from undetectable (indicating severe macular dysfunction) in a large minority to normal (3 of 28 patients), with a higher incidence of P50 delay (Fig 5) than in many other forms of maculopathy. The PERG P50 did not correlate with BCVA, highlighting the value of objective assessment of macular function, likely to be of relevance in the selection of candidates considered amenable to possible future therapeutic interventions.

All but 3 eyes of 2 molecularly confirmed patients showed pathognomonic electroretinography features consistent with ESCS. The full-field electroretinography findings in the large majority quantified the magnitude, severity, and variability of pathognomonic electroretinography abnormalities, pertinent to diagnostic accuracy and precise phenotyping. The DA rod-specific dim flash (DA 0.01) electroretinography features were undetectable in all but 1 individual, consistent with a lack of rod function, and the delayed and simplified stronger flash (DA 3.0 and DA 10.0) electroretinography responses showed qualitative similarities to the LA 3.0 electroretinography responses. In any healthy (control) participant, the LA 30-Hz electroretinography peak-to-peak amplitude is more than the LA 3.0 electroretinography a-wave and smaller than the LA 3 electroretinography b-wave.^72^^,^^73^ The LA 30-Hz electroretinography response is smaller than the LA 3 single-flash cone electroretinography a-wave in ESCS patients, as reported previously, and relates to the minimal contribution of the (relatively slow) S-cone system to the 30-Hz flicker response.^59^^,^^60^ The current study highlights both the variability and high specificity of this feature; the LA 3 a-wave-to-LA 30-Hz electroretinography amplitude ratio was never less than 1.0, and the lowest ratios (1.0) included patients with grossly reduced electroretinography responses associated with a lower signal-to-noise ratio. Furthermore, relatively increased sensitivity to short-wavelength stimulation was observed, as demonstrated by large, delayed, and simplified S-cone electroretinography responses or S-cone electroretinography responses that were larger than the corresponding LA 3 electroretinography responses. The S-cone electroretinography responses are not required for the diagnosis of ESCS, but the high correlation with the LA 3.0 electroretinography responses is consistent with both having the same S-cone-dominated origin.

All but 3 eyes of 2 molecularly confirmed patients showed the above-mentioned pathognomonic electroretinography features, in accordance with previous reports demonstrating that only patients found to harbor mutations in *NR2E3* have pathognomonic electroretinography responses when compared with patients with retinal dystrophies unrelated to *NR2E3*.^5^ Thus, in our cohort, the presence of clinical features consistent with ESCS alongside typical electroretinography responses was deemed diagnostic for ESCS, regardless of molecular confirmation.

A comparison of multiple electroretinography component amplitudes with age suggests a low mean rate of reduction over more than 6 decades, with no evidence of worsening beyond that explained by age. This finding highlights the relative stability of peripheral retinal function in most ESCS patients and may be an important prognostic consideration for retention of peripheral retinal function. Marked interparticipant variability is evident, with some younger adults showing markedly reduced electroretinography amplitudes, highlighting the importance of detailed phenotyping and the need to manage cases individually. Peak times of the main electroretinography components are delayed but show a similar high level of stability to that in the control group. Longitudinal electroretinography data were available for 3 patients, showing relatively stable responses in 2 patients and mild reduction of both LA and DA function in 1 patient.

Four evolutionary conserved domains are identified in the NR2E3 protein, shared by the nuclear hormone receptor family: the highly variable A/B domain, the n-terminal DNA binding domain, a flexible hinge region, and the ligand-binding and dimerization domain in the C terminus.^74^ Most mutations are located within the DNA binding domain and ligand-binding domain.^2^^,^^3^^,^^5^^,^^16^^,^^26^

Genetic heterogeneity occurs in ESCS. Autosomal-recessive variants in the neural retina leucine zipper (*NRL*) gene have been identified in patients demonstrating an ESCS-like phenotype.^7^^,^^75^, ^76^, ^77^, ^78^ This gene had been proposed as a possible candidate after the phenotypical characterization of the Nrl^–/–^ mouse, which revealed a complete loss of rod function and supernormal cone function, driven by overexpressed S-cones.^79^ Expression of *NR2E3* is almost absent in the Nrl^–/–^, implying that *NR2E3* is completely dependent of *NRL* expression.^18^ *NRL* encodes a basic-motif leucine zipper DNA binding protein that interacts with the paired-type homeobox transcription factor cone-rod homeobox (*CRX*) and *NR2E3*, driving the differentiation of postmitotic photoreceptors into the rod lineage.^76^, ^77^, ^78^^,^^80^, ^81^, ^82^

The function of genetic modifiers of *NR2E3*, such as the nuclear hormone receptor *Nr1d1* (Rev-erbα), has been explored as a therapeutic option in the *NR2E3*-associated retinal disease, rd7, mouse model.^26^^,^^69^^,^^83^ Delivery of the *Nr1d1* gene restored the retinal topography of the NR2E3^rd7/rd7^ neonates and reregulated the expression of key genes involved in phototransduction.^26^ Future studies will need to assess whether this approach is suited for patients with advanced disease.

The present study described the detailed clinical, imaging, molecular, and electrophysiologic findings in a cohort of 56 patients with ESCS, which, to the best of our knowledge, is the largest cohort to date. Four novel *NR2E3* variants were identified. The data quantify diagnostic electroretinography criteria and phenotypic spectrum, with evidence to suggest relative stability of peripheral retinal function over more than 6 decades, and additional evidence suggesting that central visual function remains relatively stable in most patients, which is invaluable for counseling on prognosis. Any future intervention directed at preventing visual decline in ESCS will need to address its impact on the development of macular complications, namely, foveomacular schisis and macular atrophy, which are largely responsible for the poor visual outcome observed in a subset of affected patients.
