# Supplementary material for: Enhanced S-Cone Syndrome: Spectrum of Clinical, Imaging, Electrophysiologic, and Genetic Findings in a Retrospective Case Series of 56 Patients
Source: Ophthalmol Retina. 2021 Feb;5(2):195–214. doi: 10.1016/j.oret.2020.07.008 (PMC7861019; doi:10.1016/j.oret.2020.07.008)
Supplement: Supplemental Table 1 [file mmc1.pdf]

Macular autofluorescence	*n* (RE)	Percent	Mean logMar BCVA	*n* (LE)	Percent	Mean logMar BCVA
No change	1	1.8	0.0	1	1.8	0.0
Minimal change pattern	24	42.9	0.33	20	35.7	0.30
Minimal change pattern and hyperautofluorescent flecks	10	17.9	0.46	10	17.9	0.52
Mild diffuse hypoautofluorescence	6	10.7	0.64	9	16.1	0.52
Mild diffuse hypoautofluorescence and hyperautofluorescent flecks	2	3.6	0.50	2	3.6	0.11
Moderate speckled hypoautofluorescence	6	10.7	0.37	6	10.7	0.37
Nummular (patchy) advanced hypoautofluorescence	-	-	-	1	1.8	1.77
**Peripheral autofluorescence**	**n (RE)**	**Percent**		**n (LE)**	**Percent**	
Mild diffuse hypoautofluorescence	3	7.3		3	7.3	
Mild diffuse hypoautofluorescence and hyperautofluorescent flecks	3	7.3		3	7.3	
Moderate diffuse (midperipheral half-ring or ring - <5000 μm widest diameter) hypoautofluorescence	6	14.6		6	14.6	
Moderate diffuse hypoautofluorescence + nummular (patchy) advanced hypoautofluorescence	3	7.3		3	7.3	
Moderate diffuse > 5000 μm hypoautofluorescence	3	7.3		3	7.3	
Moderate diffuse > 5000 μm hypoautofluorescence + nummular (patchy) advanced hypoautofluorescence	10	24.4		10	24.4	
Nummular (patchy) advanced hypoautofluorescence	8	19.5		8	19.5	
Hyperautofluorescent flecks	5	12.3		5	12.3	

SUPPLEMENTAL TABLE 1. Macular and peripheral patterns of fundus autofluorescence and respective mean BCVA. Abbreviations: BCVA = Best-corrected visual acuity.
