# Supplementary material for: Enhanced S-Cone Syndrome: Spectrum of Clinical, Imaging, Electrophysiologic, and Genetic Findings in a Retrospective Case Series of 56 Patients
Source: Ophthalmol Retina. 2021 Feb;5(2):195–214. doi: 10.1016/j.oret.2020.07.008 (PMC7861019; doi:10.1016/j.oret.2020.07.008)
Supplement: Supplemental Table 2 [file mmc2.pdf]

	ESCS (Right eye)		ESCS (Left eye)		Representative control		Control group 5%-95% percentile (<50yrs)	
	Peak time (ms)	Amplitude (µV)	Peak time (ms)	Amplitude (µV)	Peak time (ms)	Amplitude (µV)	Peak time (ms)	Amplitude (µV)
DA 0.01 ERG	N/A	0	N/A	0	85	420	81-108	170-370
DA 3.0 ERG<br>a-wave<br>b-wave	 27<br>62	 101<br>221	 28<br>62	 89<br>216	 16<br>56	 321<br>639	 n/a<br>n/a	 165-361<br>295-650
DA 10.0 ERG<br>a-wave<br>b-wave	 22<br>62	 167<br>290	 23<br>65	 179<br>285	 13<br>53	 390<br>655	 10-13<br>43-55	 256-434<br>373-656
LA30Hz ERG	35	32	34	28	26	144	23-27	79-185
LA 3.0 ERG<br>a-wave<br>b-wave	 20<br>41	 31<br>71	 20<br>41	 21<br>69	 14<br>29	 53<br>184	 12-14<br>26-30	 28-57<br>121-241
S-cone ERG	60	73	59	54	43	33	40-46	14-48
PERG P50	60	2.3	58	2.1	55	2.2	46-55	2.3-5.6

SUPPLEMENTAL TABLE 2. Measurements of the main ERG components of a patient with Enhanced S-Cone Syndrome (ESCS) compared with normative controls, as shown in Figure 5.
