# Supplementary material for: Enhanced S-Cone Syndrome: Spectrum of Clinical, Imaging, Electrophysiologic, and Genetic Findings in a Retrospective Case Series of 56 Patients
Source: Ophthalmol Retina. 2021 Feb;5(2):195–214. doi: 10.1016/j.oret.2020.07.008 (PMC7861019; doi:10.1016/j.oret.2020.07.008)
Supplement: Supplemental Fig 1 [file mmc3.pdf]

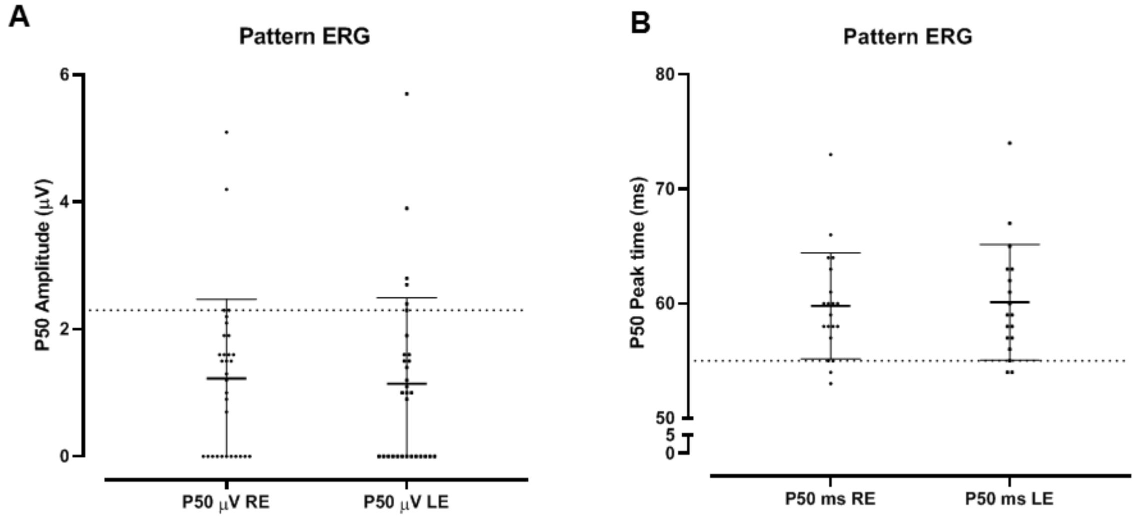

SUPPLEMENTAL FIGURE 1

Plot of pattern ERG (PERG) P50 amplitude (A) and peak time (B) responses [mean and standard deviation (SD)] in the right eye (RE) and left eye (LE) of patients with ESCS. The mean P50 amplitude was 1.2 µV (SD = 1.2) in the right eye and 1.1 µV (SD = 1.3) in the left eye and the mean P50 peak time was 59.8 ms (SD = 4.6) in the right eye and 60.11 ms (SD = 5.1) in the left eye. The 5th and 95th percentile values for the PERG P50 minimum amplitude/maximum peak time in the control group were 2.3 µV/55 ms (dashed lines).
