# Supplementary material for: Enhanced S-Cone Syndrome: Spectrum of Clinical, Imaging, Electrophysiologic, and Genetic Findings in a Retrospective Case Series of 56 Patients
Source: Ophthalmol Retina. 2021 Feb;5(2):195–214. doi: 10.1016/j.oret.2020.07.008 (PMC7861019; doi:10.1016/j.oret.2020.07.008)
Supplement: Supplemental Fig 2 [file mmc4.pdf]

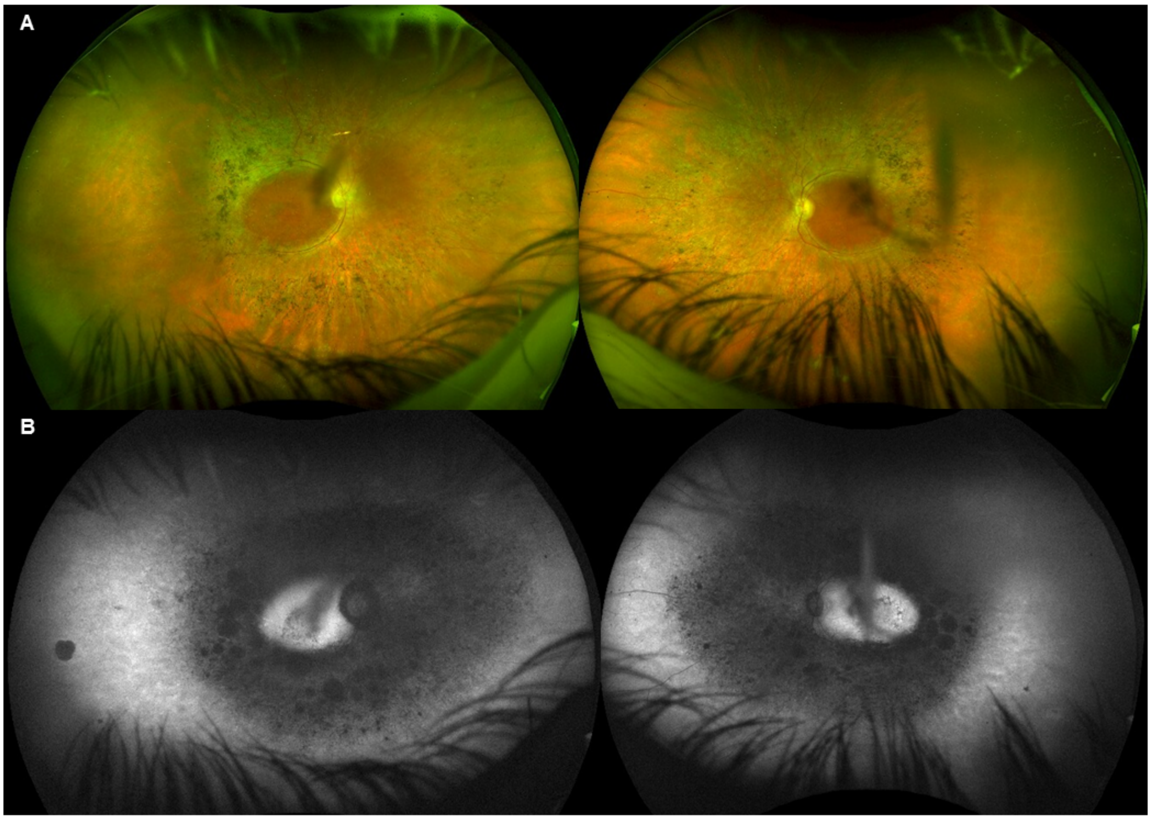


SUPPLEMENTAL FIGURE 2

Wide-field colour (A) and autofluorescence (B) fundus photographs of patient 36 with molecularly confirmed Enhanced S-Cone Syndrome (ESCS) and typical fundus features characterized by mid-peripheral white dots and nummular pigmentation. This patient had undetectable cone and rod ERG responses at age 54.
